# Supplementary material for: Patterns in evolutionary origins of heme, chlorophyll a and isopentenyl diphosphate biosynthetic pathways suggest non-photosynthetic periods prior to plastid replacements in dinoflagellates
Source: PeerJ. 2018 Aug 3;6:e5345. doi: 10.7717/peerj.5345 (PMC6078071; doi:10.7717/peerj.5345)
Supplement: Supplemental Information 1 — The details of the figures are same as those of Fig. 2, but full sequence names and their taxonomic classifications are indicated. [file peerj-06-5345-s001.pdf]

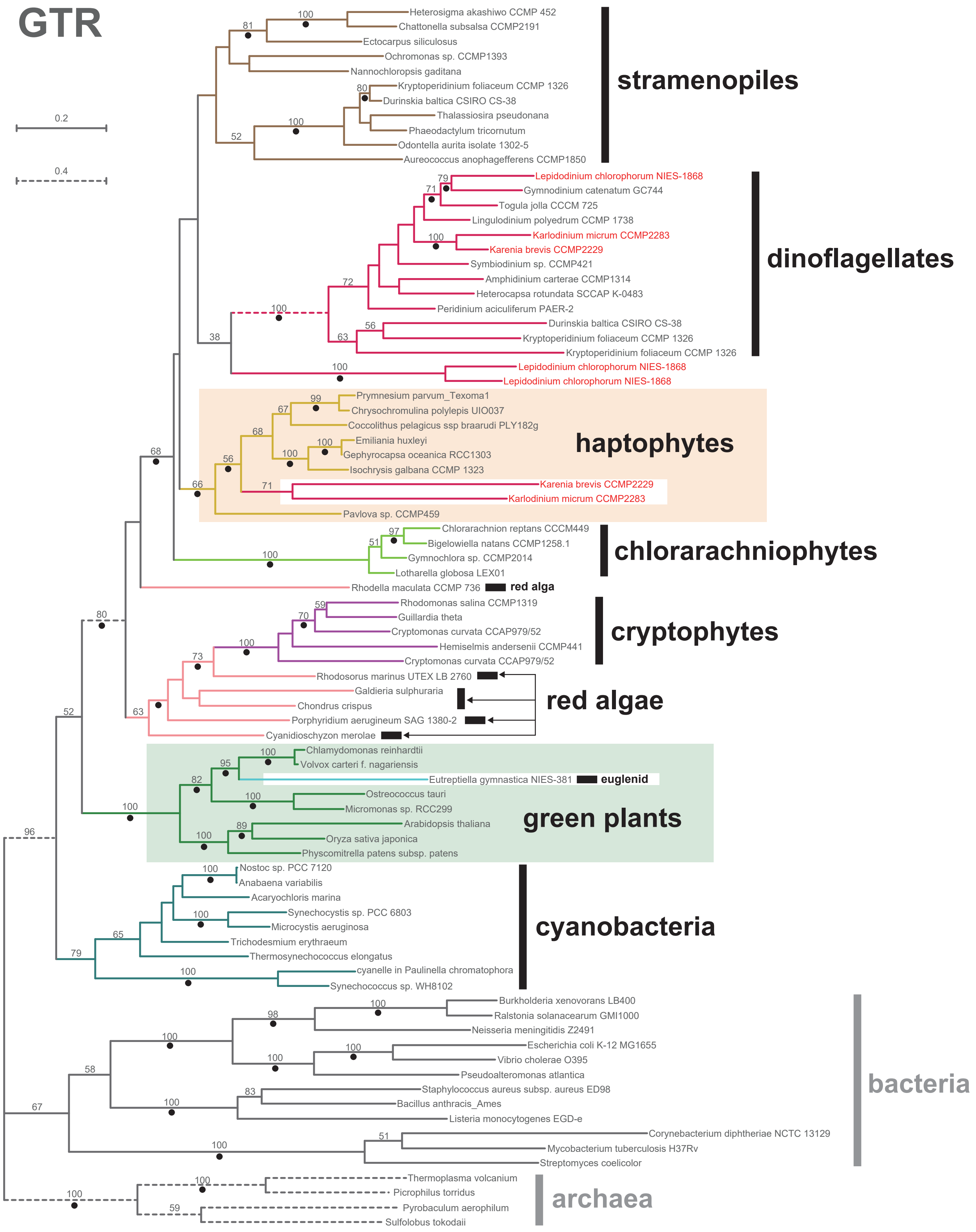
GTR
0.2
0.4
Heterosigma akashiwo CCMP 452
Chattonella subsalsa CCMP2191
Ectocarpus siliculosus
Ochromonas sp. CCMP1393
Nannochloropsis gaditana
Kryptoperidinium foliaceum CCMP 1326
Durinskia baltica CSIRO CS-38
Thalassiosira pseudonana
Phaeodactylum tricornutum
Odontella aurita isolate 1302-5
Aureococcus anophagefferens CCMP1850
stramenopiles
Lepidodinium chlorophorum NIES-1868
Gymnodinium catenatum GC744
Togula jolla CCCM 725
Lingulodinium polyedrum CCMP 1738
Karlodinium micrum CCMP2283
Karenia brevis CCMP2229
Symbiodinium sp. CCMP421
Amphidinium carterae CCMP1314
Heterocapsa rotundata SCCAP K-0483
Peridinium aciculiferum PAER-2
Durinskia baltica CSIRO CS-38
Kryptoperidinium foliaceum CCMP 1326
Kryptoperidinium foliaceum CCMP 1326
Lepidodinium chlorophorum NIES-1868
Lepidodinium chlorophorum NIES-1868
dinoflagellates
Prymnesium parvum_Texoma1
Chrysochromulina polylepis UIO037
Coccolithus pelagicus ssp braarudi PLY182g
Emiliania huxleyi
Gephyrocapsa oceanica RCC1303
Isochrysis galbana CCMP 1323
Karenia brevis CCMP2229
Karlodinium micrum CCMP2283
Pavlova sp. CCMP459
haptophytes
Chlorarachnion reptans CCCM449
Bigelowiella natans CCMP1258.1
Gymnochlora sp. CCMP2014
Lotharella globosa LEX01
chlorarachniophytes
Rhodella maculata CCMP 736
red alga
Rhodomonas salina CCMP1319
Guillardia theta
Cryptomonas curvata CCAP979/52
Hemiselmis andersenii CCMP441
Cryptomonas curvata CCAP979/52
cryptophytes
Rhodosorus marinus UTEX LB 2760
Galdieria sulphuraria
Chondrus crispus
Porphyridium aerugineum SAG 1380-2
Cyanidioschyzon merolae
red algae
Chlamydomonas reinhardtii
Volvox carteri f. nagariensis
Eutreptiella gymnastica NIES-381
euglenid
Ostreococcus tauri
Micromonas sp. RCC299
Arabidopsis thaliana
Oryza sativa japonica
Physcomitrella patens subsp. patens
green plants
Nostoc sp. PCC 7120
Anabaena variabilis
Acaryochloris marina
Synechocystis sp. PCC 6803
Microcystis aeruginosa
Trichodesmium erythraeum
Thermosynechococcus elongatus
cyanelle in Paulinella chromatophora
Synechococcus sp. WH8102
cyanobacteria
Burkholderia xenovorans LB400
Ralstonia solanacearum GMI1000
Neisseria meningitidis Z2491
Escherichia coli K-12 MG1655
Vibrio cholerae O395
Pseudoalteromonas atlantica
Staphylococcus aureus subsp. aureus ED98
Bacillus anthracis_Ames
Listeria monocytogenes EGD-e
Corynebacterium diphtheriae NCTC 13129
Mycobacterium tuberculosis H37Rv
Streptomyces coelicolor
bacteria
Thermoplasma volcanium
Picrophilus torridus
Pyrobaculum aerophilum
Sulfolobus tokodaii
archaea

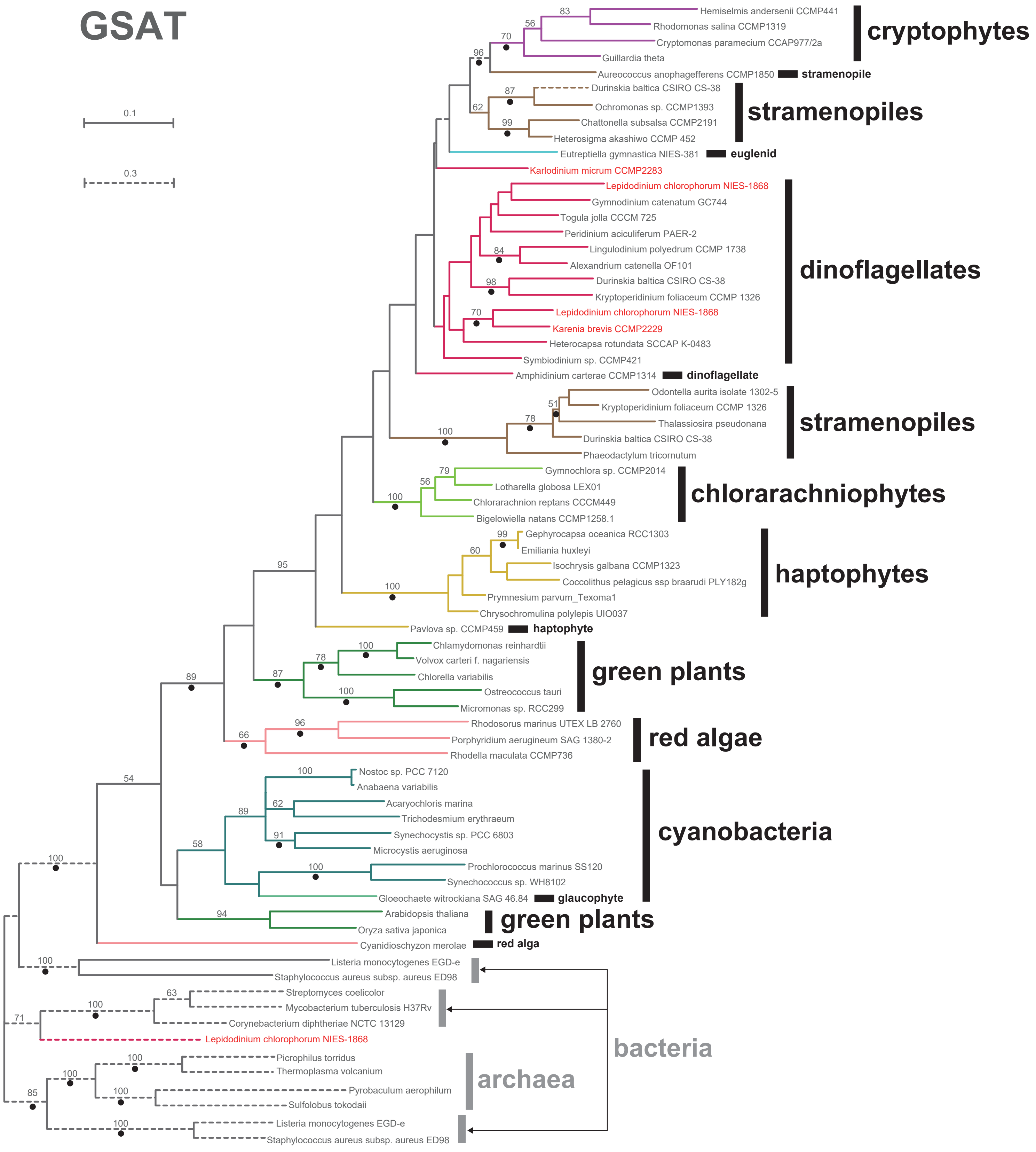
GSAT
0.1
0.3
Hemiselmis andersenii CCMP441
Rhodomonas salina CCMP1319
Cryptomonas paramecium CCAP977/2a
Guillardia theta
cryptophytes
Aureococcus anophagefferens CCMP1850
stramenopile
Durinskia baltica CSIRO CS-38
Ochromonas sp. CCMP1393
Chattonella subsalsa CCMP2191
Heterosigma akashiwo CCMP 452
stramenopiles
Eutreptiella gymnastica NIES-381
euglenid
Karlodinium micrum CCMP2283
Lepidodinium chlorophorum NIES-1868
Gymnodinium catenatum GC744
Togula jolla CCCM 725
Peridinium aciculiferum PAER-2
Lingulodinium polyedrum CCMP 1738
Alexandrium catenella OF101
Durinskia baltica CSIRO CS-38
Kryptoperidinium foliaceum CCMP 1326
Lepidodinium chlorophorum NIES-1868
Karenia brevis CCMP2229
Heterocapsa rotundata SCCAP K-0483
Symbiodinium sp. CCMP421
dinoflagellates
Amphidinium carterae CCMP1314
dinoflagellate
Odontella aurita isolate 1302-5
Kryptoperidinium foliaceum CCMP 1326
Thalassiosira pseudonana
Durinskia baltica CSIRO CS-38
Phaeodactylum tricornutum
stramenopiles
Gymnochlora sp. CCMP2014
Lotharella globosa LEX01
Chlorarachnion reptans CCCM449
Bigelowiella natans CCMP1258.1
chlorarachniophytes
Gephyrocapsa oceanica RCC1303
Emiliania huxleyi
Isochrysis galbana CCMP1323
Coccolithus pelagicus ssp braarudi PLY182g
Prymnesium parvum_Texoma1
Chrysochromulina polylepis UIO037
haptophytes
Pavlova sp. CCMP459
haptophyte
Chlamydomonas reinhardtii
Volvox carteri f. nagariensis
Chlorella variabilis
Ostreococcus tauri
Micromonas sp. RCC299
green plants
Rhodosorus marinus UTEX LB 2760
Porphyridium aerugineum SAG 1380-2
Rhodella maculata CCMP736
red algae
Nostoc sp. PCC 7120
Anabaena variabilis
Acaryochloris marina
Trichodesmium erythraeum
Synechocystis sp. PCC 6803
Microcystis aeruginosa
Prochlorococcus marinus SS120
Synechococcus sp. WH8102
Gloeochaete witrockiana SAG 46.84
glaucophyte
cyanobacteria
Arabidopsis thaliana
Oryza sativa japonica
green plants
Cyanidioschyzon merolae
red alga
Listeria monocytogenes EGD-e
Staphylococcus aureus subsp. aureus ED98
Streptomyces coelicolor
Mycobacterium tuberculosis H37Rv
Corynebacterium diphtheriae NCTC 13129
Lepidodinium chlorophorum NIES-1868
bacteria
Picrophilus torridus
Thermoplasma volcanium
Pyrobaculum aerophilum
Sulfolobus tokodaii
archaea
Listeria monocytogenes EGD-e
Staphylococcus aureus subsp. aureus ED98

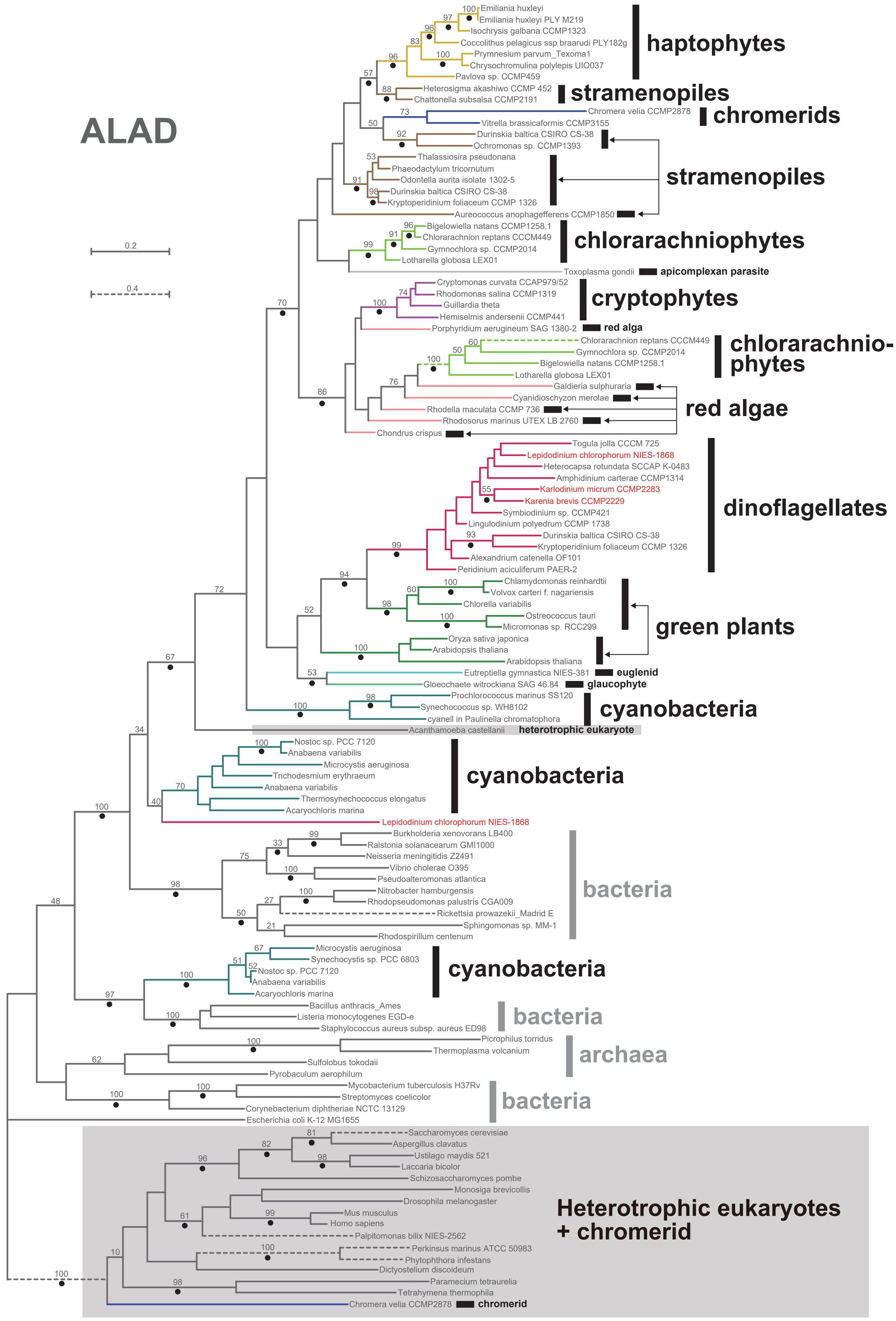

ALAD
0.2
0.4
Emiliania huxleyi
Emiliania huxleyi PLY M219
Isochrysis galbana CCMP1323
Coccolithus pelagicus ssp braarudi PLY182g
Prymnesium parvum_Texoma1
Chrysochromulina polylepis UIO037
Pavlova sp. CCMP459
haptophytes
Heterosigma akashiwo CCMP 452
Chattonella subsalsa CCMP2191
stramenopiles
Chromera velia CCMP2878
Vitrella brassicaformis CCMP3155
chromerids
Durinskia baltica CSIRO CS-38
Ochromonas sp. CCMP1393
Thalassiosira pseudonana
Phaeodactylum tricornutum
Odontella aurita isolate 1302-5
Durinskia baltica CSIRO CS-38
Kryptoperidinium foliaceum CCMP 1326
Aureococcus anophagefferens CCMP1850
stramenopiles
Bigelowiella natans CCMP1258.1
Chlorarachnion reptans CCCM449
Gymnochlora sp. CCMP2014
Lotharella globosa LEX01
chlorarachniophytes
Toxoplasma gondii
apicomplexan parasite
Cryptomonas curvata CCAP979/52
Rhodomonas salina CCMP1319
Guillardia theta
Hemiselmis andersenii CCMP441
cryptophytes
Porphyridium aerugineum SAG 1380-2
red alga
Chlorarachnion reptans CCCM449
Gymnochlora sp. CCMP2014
Bigelowiella natans CCMP1258.1
Lotharella globosa LEX01
chlorarachnio-phytes
Galdieria sulphuraria
Cyanidioschyzon merolae
Rhodella maculata CCMP 736
Rhodosorus marinus UTEX LB 2760
Chondrus crispus
red algae
Togula jolla CCCM 725
Lepidodinium chlorophorum NIES-1868
Heterocapsa rotundata SCCAP K-0483
Amphidinium carterae CCMP1314
Karlodinium micrum CCMP2283
Karenia brevis CCMP2229
Symbiodinium sp. CCMP421
Lingulodinium polyedrum CCMP 1738
Durinskia baltica CSIRO CS-38
Kryptoperidinium foliaceum CCMP 1326
Alexandrium catenella OF101
Peridinium aciculiferum PAER-2
dinoflagellates
Chlamydomonas reinhardtii
Volvox carteri f. nagariensis
Chlorella variabilis
Ostreococcus tauri
Micromonas sp. RCC299
Oryza sativa japonica
Arabidopsis thaliana
Arabidopsis thaliana
green plants
Eutreptiella gymnastica NIES-381
euglenid
Gloeochaete witrockiana SAG 46.84
glaucophyte
Prochlorococcus marinus SS120
Synechococcus sp. WH8102
cyanell in Paulinella chromatophora
cyanobacteria
Acanthamoeba castellanii
heterotrophic eukaryote
Nostoc sp. PCC 7120
Anabaena variabilis
Microcystis aeruginosa
Trichodesmium erythraeum
Anabaena variabilis
Thermosynechococcus elongatus
Acaryochloris marina
cyanobacteria
Lepidodinium chlorophorum NIES-1868
Burkholderia xenovorans LB400
Ralstonia solanacearum GMI1000
Neisseria meningitidis Z2491
Vibrio cholerae O395
Pseudoalteromonas atlantica
Nitrobacter hamburgensis
Rhodopseudomonas palustris CGA009
Rickettsia prowazekii_Madrid E
Sphingomonas sp. MM-1
Rhodospirillum centenum
bacteria
Microcystis aeruginosa
Synechocystis sp. PCC 6803
Nostoc sp. PCC 7120
Anabaena variabilis
Acaryochloris marina
cyanobacteria
Bacillus anthracis_Ames
Listeria monocytogenes EGD-e
Staphylococcus aureus subsp. aureus ED98
bacteria
Picrophilus torridus
Thermoplasma volcanium
Sulfolobus tokodaii
Pyrobaculum aerophilum
archaea
Mycobacterium tuberculosis H37Rv
Streptomyces coelicolor
Corynebacterium diphtheriae NCTC 13129
bacteria
Escherichia coli K-12 MG1655
Saccharomyces cerevisiae
Aspergillus clavatus
Ustilago maydis 521
Laccaria bicolor
Schizosaccharomyces pombe
Monosiga brevicollis
Drosophila melanogaster
Mus musculus
Homo sapiens
Palpitomonas bilix NIES-2562
Perkinsus marinus ATCC 50983
Phytophthora infestans
Dictyostelium discoideum
Paramecium tetraurelia
Tetrahymena thermophila
Chromera velia CCMP2878
chromerid
Heterotrophic eukaryotes + chromerid

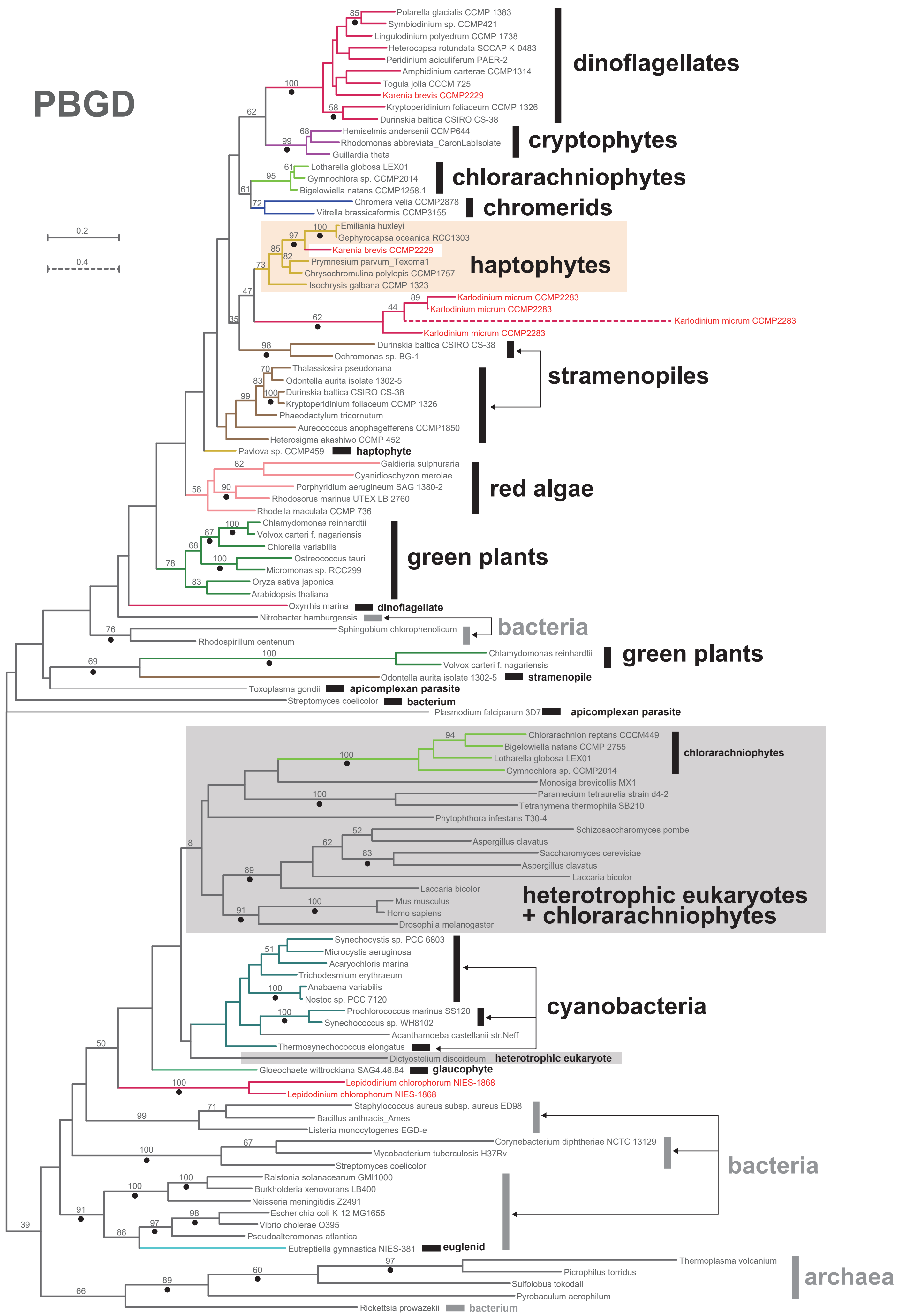

PBGD
0.2
0.4
Polarella glacialis CCMP 1383
Symbiodinium sp. CCMP421
Lingulodinium polyedrum CCMP 1738
Heterocapsa rotundata SCCAP K-0483
Peridinium aciculiferum PAER-2
Amphidinium carterae CCMP1314
Togula jolla CCCM 725
Karenia brevis CCMP2229
Kryptoperidinium foliaceum CCMP 1326
Durinskia baltica CSIRO CS-38
dinoflagellates
Hemiselmis andersenii CCMP644
Rhodomonas abbreviata_CaronLabIsolate
Guillardia theta
cryptophytes
Lotharella globosa LEX01
Gymnochlora sp. CCMP2014
Bigelowiella natans CCMP1258.1
chlorarachniophytes
Chromera velia CCMP2878
Vitrella brassicaformis CCMP3155
chromerids
Emiliania huxleyi
Gephyrocapsa oceanica RCC1303
Karenia brevis CCMP2229
Prymnesium parvum_Texoma1
Chrysochromulina polylepis CCMP1757
Isochrysis galbana CCMP 1323
haptophytes
Karlodinium micrum CCMP2283
Karlodinium micrum CCMP2283
Karlodinium micrum CCMP2283
Karlodinium micrum CCMP2283
Durinskia baltica CSIRO CS-38
Ochromonas sp. BG-1
stramenopiles
Thalassiosira pseudonana
Odontella aurita isolate 1302-5
Durinskia baltica CSIRO CS-38
Kryptoperidinium foliaceum CCMP 1326
Phaeodactylum tricornutum
Aureococcus anophagefferens CCMP1850
Heterosigma akashiwo CCMP 452
Pavlova sp. CCMP459
haptophyte
Galdieria sulphuraria
Cyanidioschyzon merolae
Porphyridium aerugineum SAG 1380-2
Rhodosorus marinus UTEX LB 2760
Rhodella maculata CCMP 736
red algae
Chlamydomonas reinhardtii
Volvox carteri f. nagariensis
Chlorella variabilis
Ostreococcus tauri
Micromonas sp. RCC299
Oryza sativa japonica
Arabidopsis thaliana
green plants
Oxyrrhis marina
dinoflagellate
Nitrobacter hamburgensis
Sphingobium chlorophenolicum
bacteria
Rhodospirillum centenum
Chlamydomonas reinhardtii
Volvox carteri f. nagariensis
green plants
Odontella aurita isolate 1302-5
stramenopile
Toxoplasma gondii
apicomplexan parasite
Streptomyces coelicolor
bacterium
Plasmodium falciparum 3D7
apicomplexan parasite
Chlorarachnion reptans CCCM449
Bigelowiella natans CCMP 2755
Lotharella globosa LEX01
Gymnochlora sp. CCMP2014
chlorarachniophytes
Monosiga brevicollis MX1
Paramecium tetraurelia strain d4-2
Tetrahymena thermophila SB210
Phytophthora infestans T30-4
Schizosaccharomyces pombe
Aspergillus clavatus
Saccharomyces cerevisiae
Aspergillus clavatus
Laccaria bicolor
Laccaria bicolor
Mus musculus
Homo sapiens
Drosophila melanogaster
heterotrophic eukaryotes
+ chlorarachniophytes
Synechocystis sp. PCC 6803
Microcystis aeruginosa
Acaryochloris marina
Trichodesmium erythraeum
Anabaena variabilis
Nostoc sp. PCC 7120
Prochlorococcus marinus SS120
Synechococcus sp. WH8102
Acanthamoeba castellanii str.Neff
Thermosynechococcus elongatus
cyanobacteria
Dictyostelium discoideum
heterotrophic eukaryote
Gloeochaete wittrockiana SAG4.46.84
glaucophyte
Lepidodinium chlorophorum NIES-1868
Lepidodinium chlorophorum NIES-1868
Staphylococcus aureus subsp. aureus ED98
Bacillus anthracis_Ames
Listeria monocytogenes EGD-e
Corynebacterium diphtheriae NCTC 13129
Mycobacterium tuberculosis H37Rv
Streptomyces coelicolor
bacteria
Ralstonia solanacearum GMI1000
Burkholderia xenovorans LB400
Neisseria meningitidis Z2491
Escherichia coli K-12 MG1655
Vibrio cholerae O395
Pseudoalteromonas atlantica
Eutreptiella gymnastica NIES-381
euglenid
Thermoplasma volcanium
Picrophilus torridus
Sulfolobus tokodaii
Pyrobaculum aerophilum
archaea
Rickettsia prowazekii
bacterium

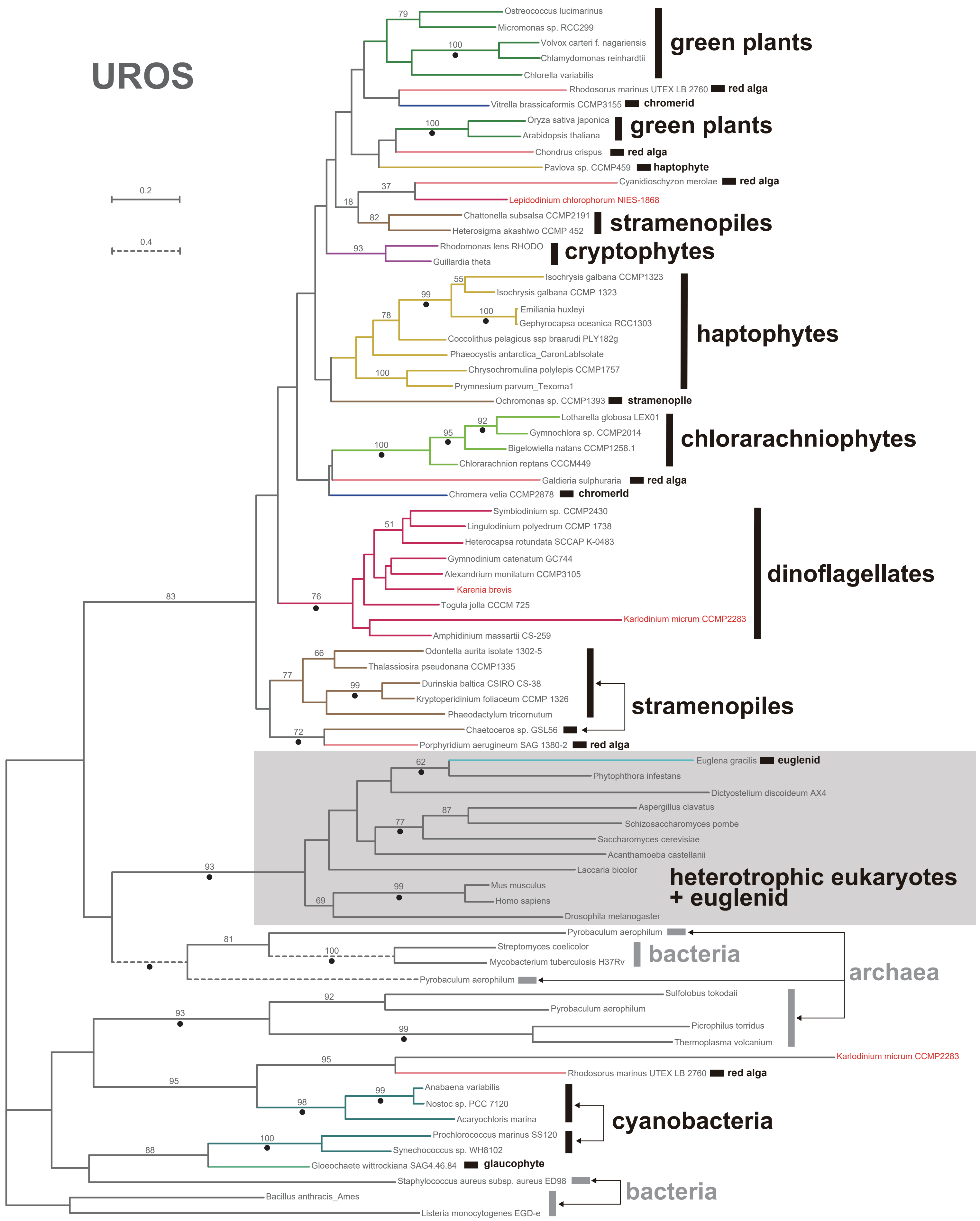

UROS
0.2
0.4
Ostreococcus lucimarinus
Micromonas sp. RCC299
Volvox carteri f. nagariensis
Chlamydomonas reinhardtii
Chlorella variabilis
green plants
Rhodosorus marinus UTEX LB 2760
red alga
Vitrella brassicaformis CCMP3155
chromerid
Oryza sativa japonica
Arabidopsis thaliana
green plants
Chondrus crispus
red alga
Pavlova sp. CCMP459
haptophyte
Cyanidioschyzon merolae
red alga
Lepidodinium chlorophorum NIES-1868
Chattonella subsalsa CCMP2191
Heterosigma akashiwo CCMP 452
stramenopiles
Rhodomonas lens RHODO
Guillardia theta
cryptophytes
Isochrysis galbana CCMP1323
Isochrysis galbana CCMP 1323
Emiliania huxleyi
Gephyrocapsa oceanica RCC1303
Coccolithus pelagicus ssp braarudi PLY182g
Phaeocystis antarctica_CaronLabIsolate
Chrysochromulina polylepis CCMP1757
Prymnesium parvum_Texoma1
haptophytes
Ochromonas sp. CCMP1393
stramenopile
Lotharella globosa LEX01
Gymnochlora sp. CCMP2014
Bigelowiella natans CCMP1258.1
Chlorarachnion reptans CCCM449
chlorarachniophytes
Galdieria sulphuraria
red alga
Chromera velia CCMP2878
chromerid
Symbiodinium sp. CCMP2430
Lingulodinium polyedrum CCMP 1738
Heterocapsa rotundata SCCAP K-0483
Gymnodinium catenatum GC744
Alexandrium monilatum CCMP3105
Karenia brevis
Togula jolla CCCM 725
Karlodinium micrum CCMP2283
Amphidinium massartii CS-259
dinoflagellates
Odontella aurita isolate 1302-5
Thalassiosira pseudonana CCMP1335
Durinskia baltica CSIRO CS-38
Kryptoperidinium foliaceum CCMP 1326
Phaeodactylum tricornutum
Chaetoceros sp. GSL56
stramenopiles
Porphyridium aerugineum SAG 1380-2
red alga
Euglena gracilis
euglenid
Phytophthora infestans
Dictyostelium discoideum AX4
Aspergillus clavatus
Schizosaccharomyces pombe
Saccharomyces cerevisiae
Acanthamoeba castellanii
Laccaria bicolor
Mus musculus
Homo sapiens
Drosophila melanogaster
heterotrophic eukaryotes + euglenid
Pyrobaculum aerophilum
Streptomyces coelicolor
Mycobacterium tuberculosis H37Rv
bacteria
Pyrobaculum aerophilum
archaea
Sulfolobus tokodaii
Pyrobaculum aerophilum
Picrophilus torridus
Thermoplasma volcanium
Karlodinium micrum CCMP2283
Rhodosorus marinus UTEX LB 2760
red alga
Anabaena variabilis
Nostoc sp. PCC 7120
Acaryochloris marina
Prochlorococcus marinus SS120
Synechococcus sp. WH8102
cyanobacteria
Gloeochaete wittrockiana SAG4.46.84
glaucophyte
Staphylococcus aureus subsp. aureus ED98
Bacillus anthracis_Ames
Listeria monocytogenes EGD-e
bacteria
79
100
100
37
18
82
93
55
99
100
78
100
92
95
100
51
76
83
66
77
99
72
62
87
77
93
99
69
81
100
92
93
99
95
95
99
98
100
88

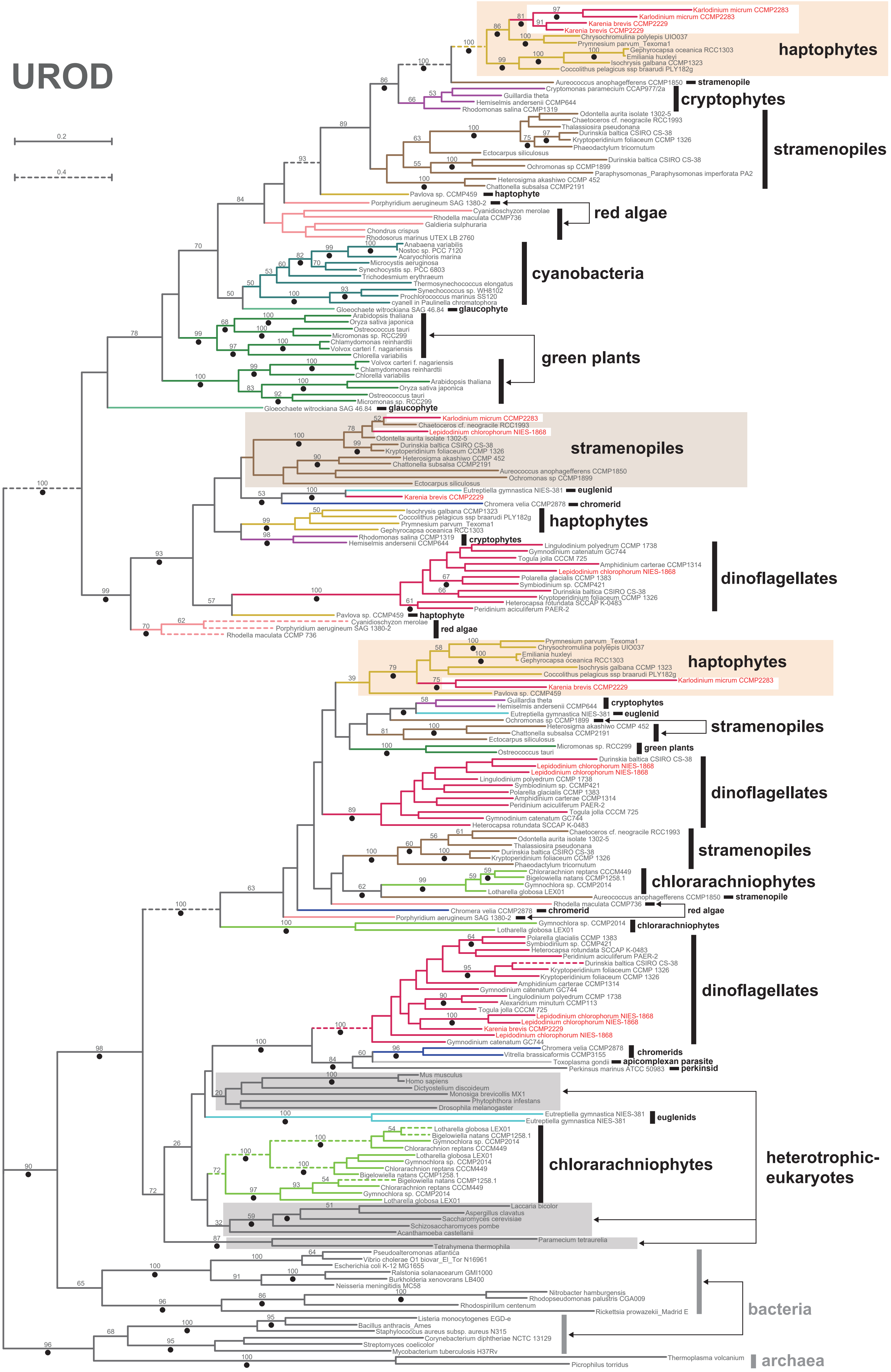
UROD
0.2
0.4
Karlodinium micrum CCMP2283
Karlodinium micrum CCMP2283
Karenia brevis CCMP2229
Karenia brevis CCMP2229
Chrysochromulina polylepis UIO037
Prymnesium parvum_Texoma1
Gephyrocapsa oceanica RCC1303
Emiliania huxleyi
Isochrysis galbana CCMP1323
Coccolithus pelagicus ssp braarudi PLY182g
haptophytes
Aureococcus anophagefferens CCMP1850
stramenopile
Cryptomonas paramecium CCAP977/2a
Guillardia theta
Hemiselmis andersenii CCMP644
Rhodomonas salina CCMP1319
cryptophytes
Odontella aurita isolate 1302-5
Chaetoceros cf. neogracile RCC1993
Thalassiosira pseudonana
Durinskia baltica CSIRO CS-38
Kryptoperidinium foliaceum CCMP 1326
Phaeodactylum tricornutum
Ectocarpus siliculosus
Durinskia baltica CSIRO CS-38
Ochromonas sp CCMP1899
Paraphysomonas_Paraphysomonas imperforata PA2
Heterosigma akashiwo CCMP 452
Chattonella subsalsa CCMP2191
stramenopiles
Pavlova sp. CCMP459
haptophyte
Porphyridium aerugineum SAG 1380-2
Cyanidioschyzon merolae
Rhodella maculata CCMP736
Galdieria sulphuraria
Chondrus crispus
Rhodosorus marinus UTEX LB 2760
red algae
Anabaena variabilis
Nostoc sp. PCC 7120
Acaryochloris marina
Microcystis aeruginosa
Synechocystis sp. PCC 6803
Trichodesmium erythraeum
Thermosynechococcus elongatus
Synechococcus sp. WH8102
Prochlorococcus marinus SS120
cyanell in Paulinella chromatophora
cyanobacteria
Gloeochaete witrockiana SAG 46.84
glaucophyte
Arabidopsis thaliana
Oryza sativa japonica
Ostreococcus tauri
Micromonas sp. RCC299
Chlamydomonas reinhardtii
Volvox carteri f. nagariensis
Chlorella variabilis
Volvox carteri f. nagariensis
Chlamydomonas reinhardtii
Chlorella variabilis
Arabidopsis thaliana
Oryza sativa japonica
Ostreococcus tauri
Micromonas sp. RCC299
green plants
Gloeochaete witrockiana SAG 46.84
glaucophyte
Karlodinium micrum CCMP2283
Chaetoceros cf. neogracile RCC1993
Lepidodinium chlorophorum NIES-1868
Odontella aurita isolate 1302-5
Durinskia baltica CSIRO CS-38
Kryptoperidinium foliaceum CCMP 1326
Heterosigma akashiwo CCMP 452
Chattonella subsalsa CCMP2191
Aureococcus anophagefferens CCMP1850
Ochromonas sp CCMP1899
Ectocarpus siliculosus
stramenopiles
Eutreptiella gymnastica NIES-381
euglenid
Karenia brevis CCMP2229
Chromera velia CCMP2878
chromerid
Isochrysis galbana CCMP1323
Coccolithus pelagicus ssp braarudi PLY182g
Prymnesium parvum_Texoma1
Gephyrocapsa oceanica RCC1303
haptophytes
Rhodomonas salina CCMP1319
Hemiselmis andersenii CCMP644
cryptophytes
Lingulodinium polyedrum CCMP 1738
Gymnodinium catenatum GC744
Togula jolla CCCM 725
Amphidinium carterae CCMP1314
Lepidodinium chlorophorum NIES-1868
Polarella glacialis CCMP 1383
Symbiodinium sp. CCMP421
Durinskia baltica CSIRO CS-38
Kryptoperidinium foliaceum CCMP 1326
Heterocapsa rotundata SCCAP K-0483
Peridinium aciculiferum PAER-2
dinoflagellates
Pavlova sp. CCMP459
haptophyte
Cyanidioschyzon merolae
Porphyridium aerugineum SAG 1380-2
Rhodella maculata CCMP 736
red algae
Prymnesium parvum_Texoma1
Chrysochromulina polylepis UIO037
Emiliania huxleyi
Gephyrocapsa oceanica RCC1303
Isochrysis galbana CCMP 1323
Coccolithus pelagicus ssp braarudi PLY182g
Karlodinium micrum CCMP2283
Karenia brevis CCMP2229
Pavlova sp. CCMP459
haptophytes
Guillardia theta
Hemiselmis andersenii CCMP644
cryptophytes
Eutreptiella gymnastica NIES-381
euglenid
Ochromonas sp CCMP1899
Heterosigma akashiwo CCMP 452
Chattonella subsalsa CCMP2191
Ectocarpus siliculosus
stramenopiles
Micromonas sp. RCC299
Ostreococcus tauri
green plants
Durinskia baltica CSIRO CS-38
Lepidodinium chlorophorum NIES-1868
Lepidodinium chlorophorum NIES-1868
Lingulodinium polyedrum CCMP 1738
Symbiodinium sp. CCMP421
Polarella glacialis CCMP 1383
Amphidinium carterae CCMP1314
Peridinium aciculiferum PAER-2
Togula jolla CCCM 725
Gymnodinium catenatum GC744
Heterocapsa rotundata SCCAP K-0483
dinoflagellates
Chaetoceros cf. neogracile RCC1993
Odontella aurita isolate 1302-5
Thalassiosira pseudonana
Durinskia baltica CSIRO CS-38
Kryptoperidinium foliaceum CCMP 1326
Phaeodactylum tricornutum
stramenopiles
Chlorarachnion reptans CCCM449
Bigelowiella natans CCMP1258.1
Gymnochlora sp. CCMP2014
Lotharella globosa LEX01
chlorarachniophytes
Aureococcus anophagefferens CCMP1850
stramenopile
Rhodella maculata CCMP736
red algae
Chromera velia CCMP2878
chromerid
Porphyridium aerugineum SAG 1380-2
Gymnochlora sp. CCMP2014
Lotharella globosa LEX01
chlorarachniophytes
Polarella glacialis CCMP 1383
Symbiodinium sp. CCMP421
Heterocapsa rotundata SCCAP K-0483
Peridinium aciculiferum PAER-2
Durinskia baltica CSIRO CS-38
Kryptoperidinium foliaceum CCMP 1326
Kryptoperidinium foliaceum CCMP 1326
Amphidinium carterae CCMP1314
Gymnodinium catenatum GC744
Lingulodinium polyedrum CCMP 1738
Alexandrium minutum CCMP113
Togula jolla CCCM 725
Lepidodinium chlorophorum NIES-1868
Lepidodinium chlorophorum NIES-1868
Karenia brevis CCMP2229
Lepidodinium chlorophorum NIES-1868
Gymnodinium catenatum GC744
dinoflagellates
Chromera velia CCMP2878
Vitrella brassicaformis CCMP3155
chromerids
Toxoplasma gondii
apicomplexan parasite
Perkinsus marinus ATCC 50983
perkinsid
Mus musculus
Homo sapiens
Dictyostelium discoideum
Monosiga brevicollis MX1
Phytophthora infestans
Drosophila melanogaster
Eutreptiella gymnastica NIES-381
Eutreptiella gymnastica NIES-381
euglenids
Lotharella globosa LEX01
Bigelowiella natans CCMP1258.1
Gymnochlora sp. CCMP2014
Chlorarachnion reptans CCCM449
Lotharella globosa LEX01
Gymnochlora sp. CCMP2014
Chlorarachnion reptans CCCM449
Bigelowiella natans CCMP1258.1
Bigelowiella natans CCMP1258.1
Chlorarachnion reptans CCCM449
Gymnochlora sp. CCMP2014
Lotharella globosa LEX01
chlorarachniophytes
heterotrophic-eukaryotes
Laccaria bicolor
Aspergillus clavatus
Saccharomyces cerevisiae
Schizosaccharomyces pombe
Acanthamoeba castellanii
Paramecium tetraurelia
Tetrahymena thermophila
Pseudoalteromonas atlantica
Vibrio cholerae O1 biovar_El_Tor N16961
Escherichia coli K-12 MG1655
Ralstonia solanacearum GMI1000
Burkholderia xenovorans LB400
Neisseria meningitidis MC58
Nitrobacter hamburgensis
Rhodopseudomonas palustris CGA009
Rhodospirillum centenum
Rickettsia prowazekii_Madrid E
bacteria
Listeria monocytogenes EGD-e
Bacillus anthracis_Ames
Staphylococcus aureus subsp. aureus N315
Corynebacterium diphtheriae NCTC 13129
Streptomyces coelicolor
Mycobacterium tuberculosis H37Rv
Thermoplasma volcanium
Picrophilus torridus
archaea

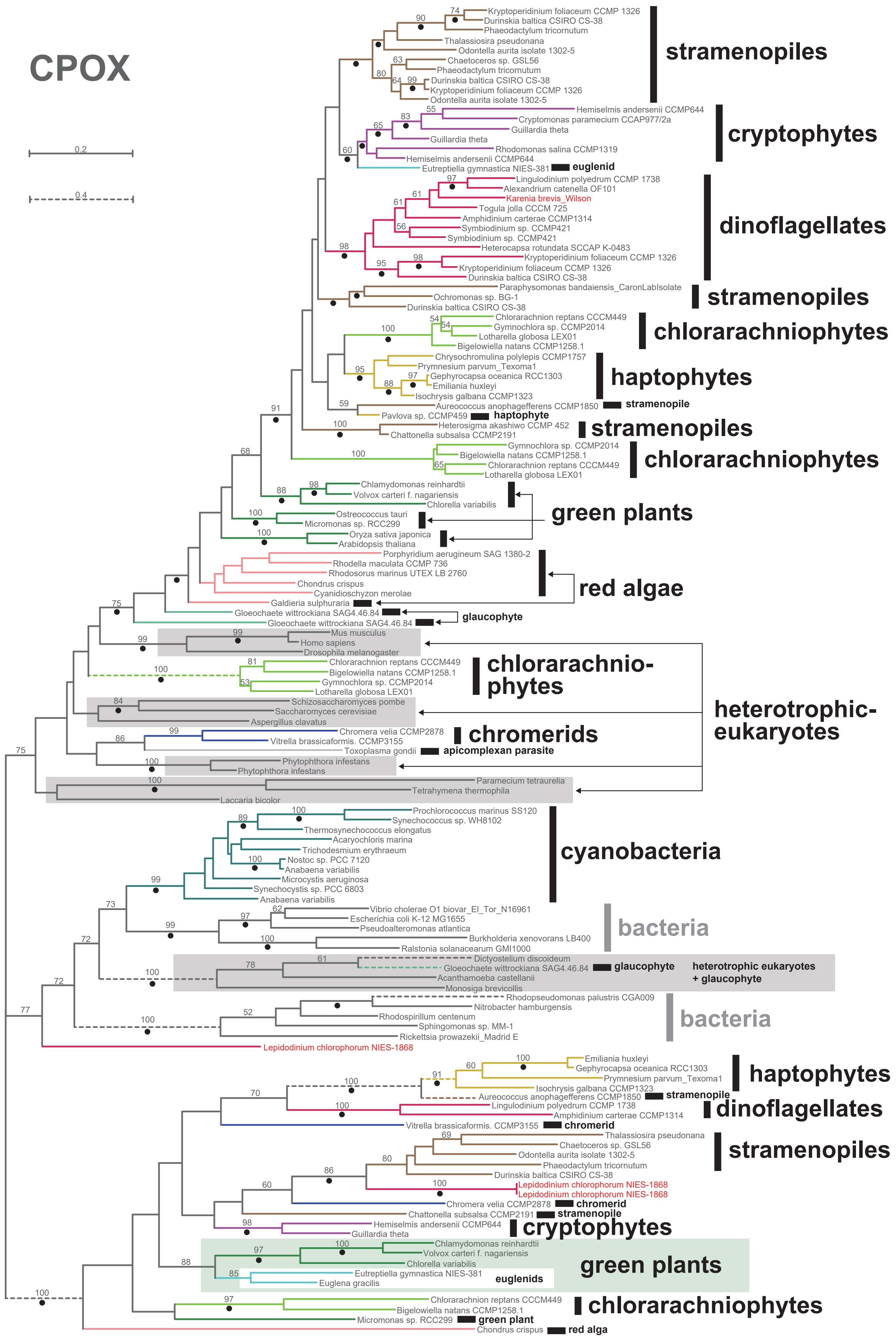

CPOX
0.2
0.4
Kryptoperidinium foliaceum CCMP 1326
Durinskia baltica CSIRO CS-38
Phaeodactylum tricornutum
Thalassiosira pseudonana
Odontella aurita isolate 1302-5
Chaetoceros sp. GSL56
Phaeodactylum tricornutum
Durinskia baltica CSIRO CS-38
Kryptoperidinium foliaceum CCMP 1326
Odontella aurita isolate 1302-5
stramenopiles
Hemiselmis andersenii CCMP644
Cryptomonas paramecium CCAP977/2a
Guillardia theta
Guillardia theta
Rhodomonas salina CCMP1319
Hemiselmis andersenii CCMP644
cryptophytes
Eutreptiella gymnastica NIES-381
euglenid
Lingulodinium polyedrum CCMP 1738
Alexandrium catenella OF101
Karenia brevis_Wilson
Togula jolla CCCM 725
Amphidinium carterae CCMP1314
Symbiodinium sp. CCMP421
Symbiodinium sp. CCMP421
Heterocapsa rotundata SCCAP K-0483
Kryptoperidinium foliaceum CCMP 1326
Kryptoperidinium foliaceum CCMP 1326
Durinskia baltica CSIRO CS-38
dinoflagellates
Paraphysomonas bandaiensis_CaronLabIsolate
Ochromonas sp. BG-1
Durinskia baltica CSIRO CS-38
stramenopiles
Chlorarachnion reptans CCCM449
Gymnochlora sp. CCMP2014
Lotharella globosa LEX01
Bigelowiella natans CCMP1258.1
chlorarachniophytes
Chrysochromulina polylepis CCMP1757
Prymnesium parvum_Texoma1
Gephyrocapsa oceanica RCC1303
Emiliania huxleyi
Isochrysis galbana CCMP1323
haptophytes
Aureococcus anophagefferens CCMP1850
stramenopile
Pavlova sp. CCMP459
haptophyte
Heterosigma akashiwo CCMP 452
Chattonella subsalsa CCMP2191
stramenopiles
Gymnochlora sp. CCMP2014
Bigelowiella natans CCMP1258.1
Chlorarachnion reptans CCCM449
Lotharella globosa LEX01
chlorarachniophytes
Chlamydomonas reinhardtii
Volvox carteri f. nagariensis
Chlorella variabilis
Ostreococcus tauri
Micromonas sp. RCC299
Oryza sativa japonica
Arabidopsis thaliana
green plants
Porphyridium aerugineum SAG 1380-2
Rhodella maculata CCMP 736
Rhodosorus marinus UTEX LB 2760
Chondrus crispus
Cyanidioschyzon merolae
Galdieria sulphuraria
red algae
Gloeochaete wittrockiana SAG4.46.84
Gloeochaete wittrockiana SAG4.46.84
glaucophyte
Mus musculus
Homo sapiens
Drosophila melanogaster
Chlorarachnion reptans CCCM449
Bigelowiella natans CCMP1258.1
Gymnochlora sp. CCMP2014
Lotharella globosa LEX01
chlorarachnio-
phytes
Schizosaccharomyces pombe
Saccharomyces cerevisiae
Aspergillus clavatus
heterotrophic-
eukaryotes
Chromera velia CCMP2878
Vitrella brassicaformis. CCMP3155
chromerids
Toxoplasma gondii
apicomplexan parasite
Phytophthora infestans
Phytophthora infestans
Paramecium tetraurelia
Tetrahymena thermophila
Laccaria bicolor
Prochlorococcus marinus SS120
Synechococcus sp. WH8102
Thermosynechococcus elongatus
Acaryochloris marina
Trichodesmium erythraeum
Nostoc sp. PCC 7120
Anabaena variabilis
Microcystis aeruginosa
Synechocystis sp. PCC 6803
Anabaena variabilis
cyanobacteria
Vibrio cholerae O1 biovar_El_Tor_N16961
Escherichia coli K-12 MG1655
Pseudoalteromonas atlantica
Burkholderia xenovorans LB400
Ralstonia solanacearum GMI1000
bacteria
Dictyostelium discoideum
Gloeochaete wittrockiana SAG4.46.84
glaucophyte
Acanthamoeba castellanii
Monosiga brevicollis
heterotrophic eukaryotes
+ glaucophyte
Rhodopseudomonas palustris CGA009
Nitrobacter hamburgensis
Rhodospirillum centenum
Sphingomonas sp. MM-1
Rickettsia prowazekii_Madrid E
bacteria
Lepidodinium chlorophorum NIES-1868
Emiliania huxleyi
Gephyrocapsa oceanica RCC1303
Prymnesium parvum_Texoma1
Isochrysis galbana CCMP1323
haptophytes
Aureococcus anophagefferens CCMP1850
stramenopile
Lingulodinium polyedrum CCMP 1738
Amphidinium carterae CCMP1314
dinoflagellates
Vitrella brassicaformis. CCMP3155
chromerid
Thalassiosira pseudonana
Chaetoceros sp. GSL56
Odontella aurita isolate 1302-5
Phaeodactylum tricornutum
Durinskia baltica CSIRO CS-38
stramenopiles
Lepidodinium chlorophorum NIES-1868
Lepidodinium chlorophorum NIES-1868
Chromera velia CCMP2878
chromerid
Chattonella subsalsa CCMP2191
stramenopile
Hemiselmis andersenii CCMP644
Guillardia theta
cryptophytes
Chlamydomonas reinhardtii
Volvox carteri f. nagariensis
Chlorella variabilis
green plants
Eutreptiella gymnastica NIES-381
Euglena gracilis
euglenids
Chlorarachnion reptans CCCM449
Bigelowiella natans CCMP1258.1
chlorarachniophytes
Micromonas sp. RCC299
green plant
Chondrus crispus
red alga

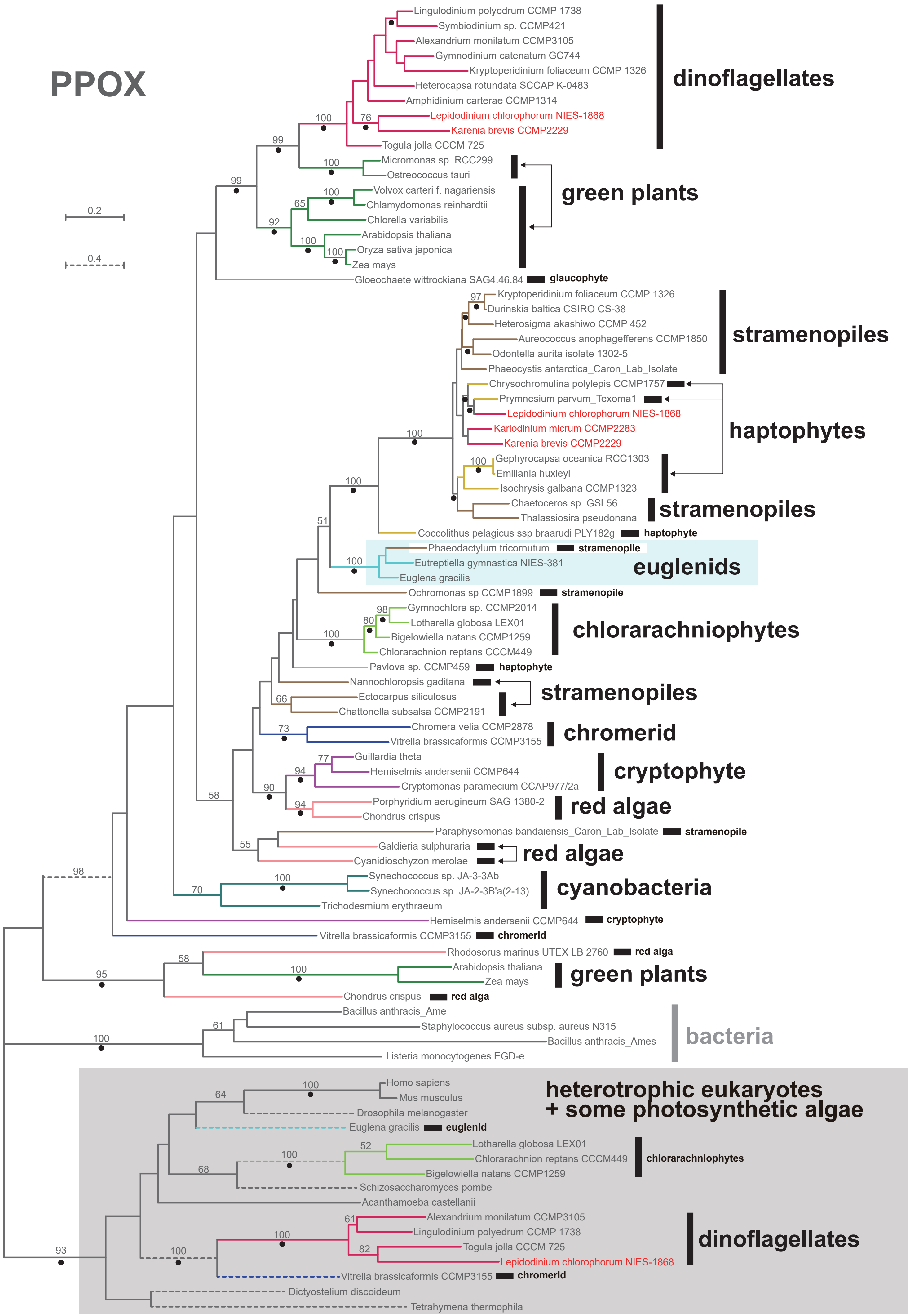
PPOX
0.2
0.4
Lingulodinium polyedrum CCMP 1738
Symbiodinium sp. CCMP421
Alexandrium monilatum CCMP3105
Gymnodinium catenatum GC744
Kryptoperidinium foliaceum CCMP 1326
Heterocapsa rotundata SCCAP K-0483
Amphidinium carterae CCMP1314
Lepidodinium chlorophorum NIES-1868
Karenia brevis CCMP2229
Togula jolla CCCM 725
dinoflagellates
Micromonas sp. RCC299
Ostreococcus tauri
Volvox carteri f. nagariensis
Chlamydomonas reinhardtii
Chlorella variabilis
Arabidopsis thaliana
Oryza sativa japonica
Zea mays
green plants
Gloeochaete wittrockiana SAG4.46.84
glaucophyte
Kryptoperidinium foliaceum CCMP 1326
Durinskia baltica CSIRO CS-38
Heterosigma akashiwo CCMP 452
Aureococcus anophagefferens CCMP1850
Odontella aurita isolate 1302-5
Phaeocystis antarctica_Caron_Lab_Isolate
stramenopiles
Chrysochromulina polylepis CCMP1757
Prymnesium parvum_Texoma1
Lepidodinium chlorophorum NIES-1868
Karlodinium micrum CCMP2283
Karenia brevis CCMP2229
haptophytes
Gephyrocapsa oceanica RCC1303
Emiliania huxleyi
Isochrysis galbana CCMP1323
Chaetoceros sp. GSL56
Thalassiosira pseudonana
stramenopiles
Coccolithus pelagicus ssp braarudi PLY182g
haptophyte
Phaeodactylum tricornutum
stramenopile
Eutreptiella gymnastica NIES-381
Euglena gracilis
euglenids
Ochromonas sp CCMP1899
stramenopile
Gymnochlora sp. CCMP2014
Lotharella globosa LEX01
Bigelowiella natans CCMP1259
Chlorarachnion reptans CCCM449
chlorarachniophytes
Pavlova sp. CCMP459
haptophyte
Nannochloropsis gaditana
Ectocarpus siliculosus
Chattonella subsalsa CCMP2191
stramenopiles
Chromera velia CCMP2878
Vitrella brassicaformis CCMP3155
chromerid
Guillardia theta
Hemiselmis andersenii CCMP644
Cryptomonas paramecium CCAP977/2a
cryptophyte
Porphyridium aerugineum SAG 1380-2
Chondrus crispus
red algae
Paraphysomonas bandaiensis_Caron_Lab_Isolate
stramenopile
Galdieria sulphuraria
Cyanidioschyzon merolae
red algae
Synechococcus sp. JA-3-3Ab
Synechococcus sp. JA-2-3B'a(2-13)
Trichodesmium erythraeum
cyanobacteria
Hemiselmis andersenii CCMP644
cryptophyte
Vitrella brassicaformis CCMP3155
chromerid
Rhodosorus marinus UTEX LB 2760
red alga
Arabidopsis thaliana
Zea mays
green plants
Chondrus crispus
red alga
Bacillus anthracis_Ame
Staphylococcus aureus subsp. aureus N315
Bacillus anthracis_Ames
Listeria monocytogenes EGD-e
bacteria
Homo sapiens
Mus musculus
Drosophila melanogaster
Euglena gracilis
euglenid
heterotrophic eukaryotes
+ some photosynthetic algae
Lotharella globosa LEX01
Chlorarachnion reptans CCCM449
Bigelowiella natans CCMP1259
chlorarachniophytes
Schizosaccharomyces pombe
Acanthamoeba castellanii
Alexandrium monilatum CCMP3105
Lingulodinium polyedrum CCMP 1738
Togula jolla CCCM 725
Lepidodinium chlorophorum NIES-1868
dinoflagellates
Vitrella brassicaformis CCMP3155
chromerid
Dictyostelium discoideum
Tetrahymena thermophila

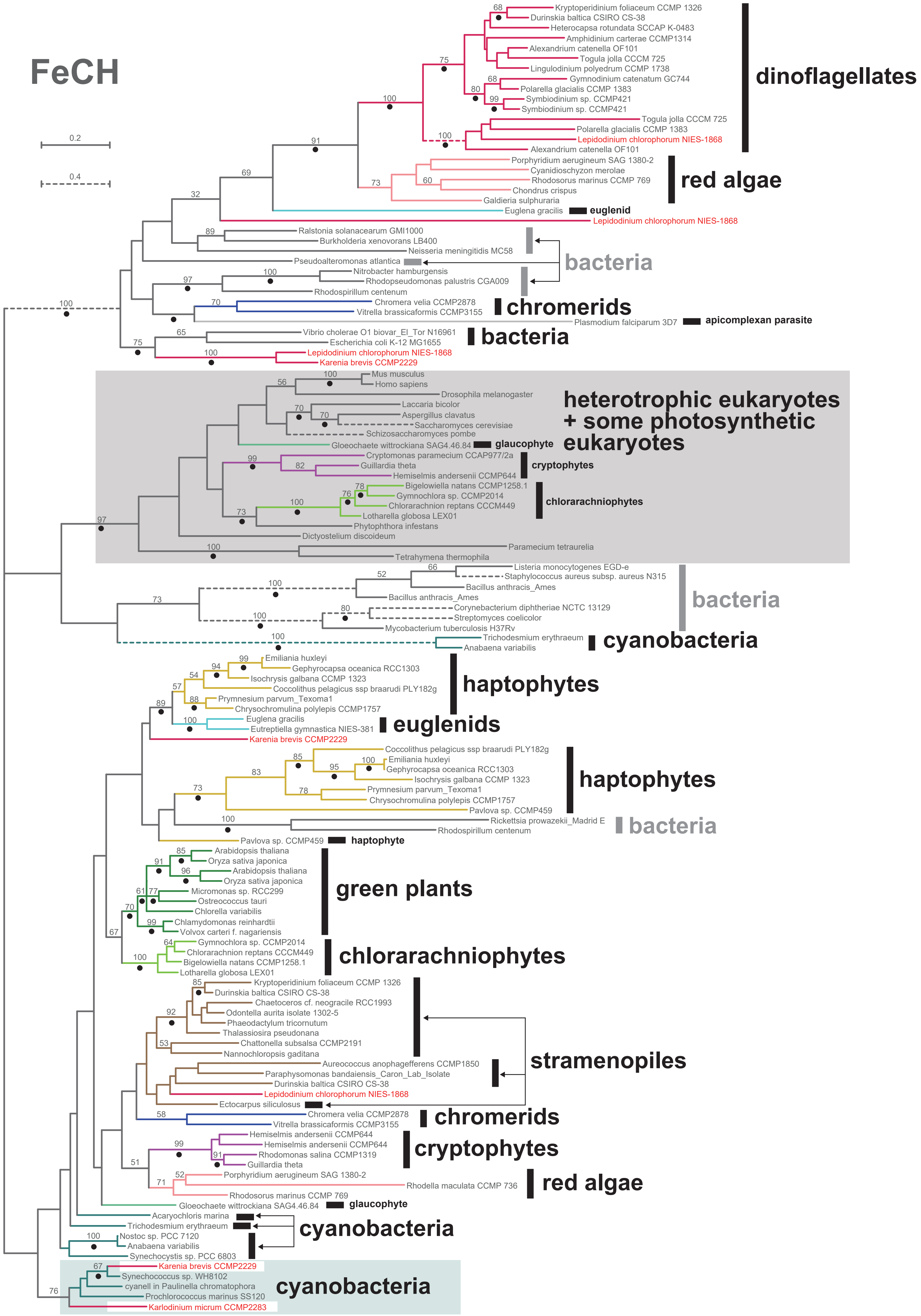
FeCH
0.2
0.4
Kryptoperidinium foliaceum CCMP 1326
Durinskia baltica CSIRO CS-38
Heterocapsa rotundata SCCAP K-0483
Amphidinium carterae CCMP1314
Alexandrium catenella OF101
Togula jolla CCCM 725
Lingulodinium polyedrum CCMP 1738
Gymnodinium catenatum GC744
Polarella glacialis CCMP 1383
Symbiodinium sp. CCMP421
Symbiodinium sp. CCMP421
Togula jolla CCCM 725
Polarella glacialis CCMP 1383
Lepidodinium chlorophorum NIES-1868
Alexandrium catenella OF101
dinoflagellates
Porphyridium aerugineum SAG 1380-2
Cyanidioschyzon merolae
Rhodosorus marinus CCMP 769
Chondrus crispus
Galdieria sulphuraria
red algae
Euglena gracilis
euglenid
Lepidodinium chlorophorum NIES-1868
Ralstonia solanacearum GMI1000
Burkholderia xenovorans LB400
Neisseria meningitidis MC58
Pseudoalteromonas atlantica
bacteria
Nitrobacter hamburgensis
Rhodopseudomonas palustris CGA009
Rhodospirillum centenum
Chromera velia CCMP2878
Vitrella brassicaformis CCMP3155
chromerids
Plasmodium falciparum 3D7
apicomplexan parasite
Vibrio cholerae O1 biovar_El_Tor N16961
Escherichia coli K-12 MG1655
bacteria
Lepidodinium chlorophorum NIES-1868
Karenia brevis CCMP2229
Mus musculus
Homo sapiens
Drosophila melanogaster
Laccaria bicolor
Aspergillus clavatus
Saccharomyces cerevisiae
Schizosaccharomyces pombe
heterotrophic eukaryotes
+ some photosynthetic
eukaryotes
Gloeochaete wittrockiana SAG4.46.84
glaucophyte
Cryptomonas paramecium CCAP977/2a
Guillardia theta
Hemiselmis andersenii CCMP644
cryptophytes
Bigelowiella natans CCMP1258.1
Gymnochlora sp. CCMP2014
Chlorarachnion reptans CCCM449
Lotharella globosa LEX01
chlorarachniophytes
Phytophthora infestans
Dictyostelium discoideum
Paramecium tetraurelia
Tetrahymena thermophila
Listeria monocytogenes EGD-e
Staphylococcus aureus subsp. aureus N315
Bacillus anthracis_Ames
Bacillus anthracis_Ames
Corynebacterium diphtheriae NCTC 13129
Streptomyces coelicolor
Mycobacterium tuberculosis H37Rv
bacteria
Trichodesmium erythraeum
Anabaena variabilis
cyanobacteria
Emiliania huxleyi
Gephyrocapsa oceanica RCC1303
Isochrysis galbana CCMP 1323
Coccolithus pelagicus ssp braarudi PLY182g
Prymnesium parvum_Texoma1
Chrysochromulina polylepis CCMP1757
haptophytes
Euglena gracilis
Eutreptiella gymnastica NIES-381
euglenids
Karenia brevis CCMP2229
Coccolithus pelagicus ssp braarudi PLY182g
Emiliania huxleyi
Gephyrocapsa oceanica RCC1303
Isochrysis galbana CCMP 1323
Prymnesium parvum_Texoma1
Chrysochromulina polylepis CCMP1757
Pavlova sp. CCMP459
haptophytes
Rickettsia prowazekii_Madrid E
Rhodospirillum centenum
bacteria
Pavlova sp. CCMP459
haptophyte
Arabidopsis thaliana
Oryza sativa japonica
Arabidopsis thaliana
Oryza sativa japonica
Micromonas sp. RCC299
Ostreococcus tauri
Chlorella variabilis
Chlamydomonas reinhardtii
Volvox carteri f. nagariensis
green plants
Gymnochlora sp. CCMP2014
Chlorarachnion reptans CCCM449
Bigelowiella natans CCMP1258.1
Lotharella globosa LEX01
chlorarachniophytes
Kryptoperidinium foliaceum CCMP 1326
Durinskia baltica CSIRO CS-38
Chaetoceros cf. neogracile RCC1993
Odontella aurita isolate 1302-5
Phaeodactylum tricornutum
Thalassiosira pseudonana
Chattonella subsalsa CCMP2191
Nannochloropsis gaditana
Aureococcus anophagefferens CCMP1850
Paraphysomonas bandaiensis_Caron_Lab_Isolate
Durinskia baltica CSIRO CS-38
Lepidodinium chlorophorum NIES-1868
Ectocarpus siliculosus
stramenopiles
Chromera velia CCMP2878
Vitrella brassicaformis CCMP3155
chromerids
Hemiselmis andersenii CCMP644
Hemiselmis andersenii CCMP644
Rhodomonas salina CCMP1319
Guillardia theta
cryptophytes
Porphyridium aerugineum SAG 1380-2
Rhodella maculata CCMP 736
Rhodosorus marinus CCMP 769
red algae
Gloeochaete wittrockiana SAG4.46.84
glaucophyte
Acaryochloris marina
Trichodesmium erythraeum
Nostoc sp. PCC 7120
Anabaena variabilis
Synechocystis sp. PCC 6803
cyanobacteria
Karenia brevis CCMP2229
Synechococcus sp. WH8102
cyanell in Paulinella chromatophora
Prochlorococcus marinus SS120
Karlodinium micrum CCMP2283
cyanobacteria
