# Supplementary figures and images for: Patterns in evolutionary origins of heme, chlorophyll a and isopentenyl diphosphate biosynthetic pathways suggest non-photosynthetic periods prior to plastid replacements in dinoflagellates
Source: PeerJ. 2018 Aug 3;6:e5345. doi: 10.7717/peerj.5345 (PMC6078071; doi:10.7717/peerj.5345)

# MgCH(ChID)

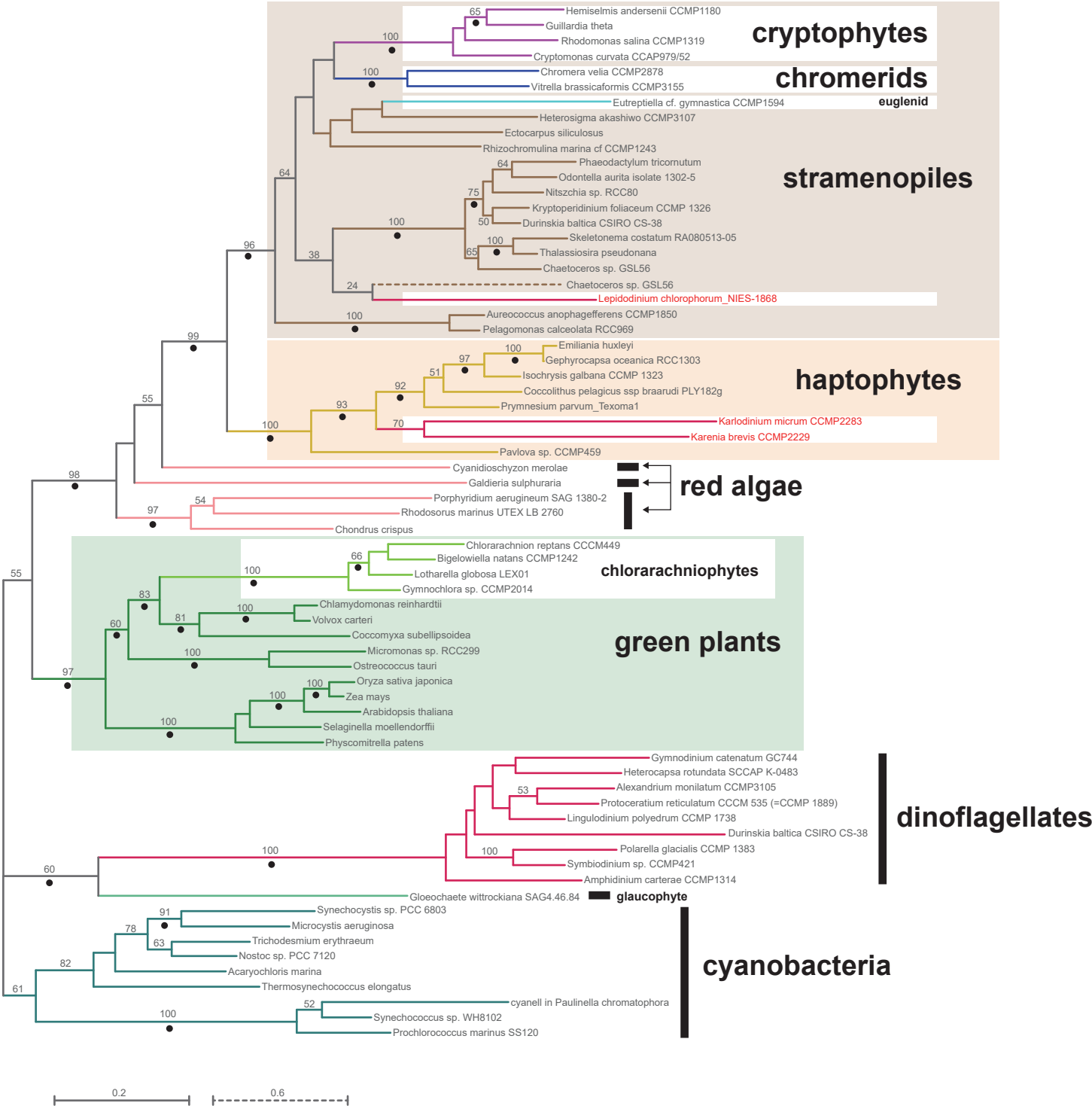

MgCH(ChlH)

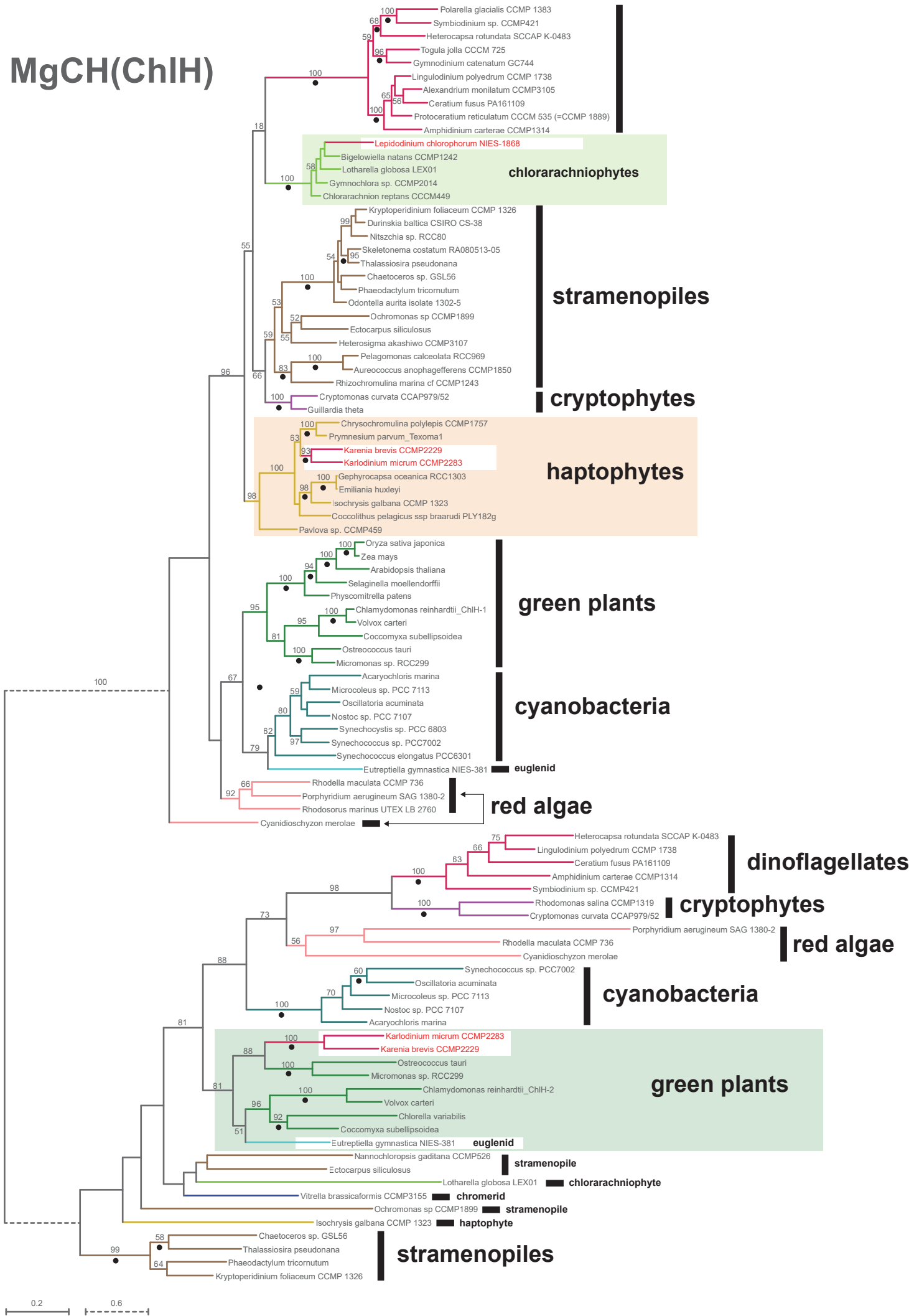

# MgPMT

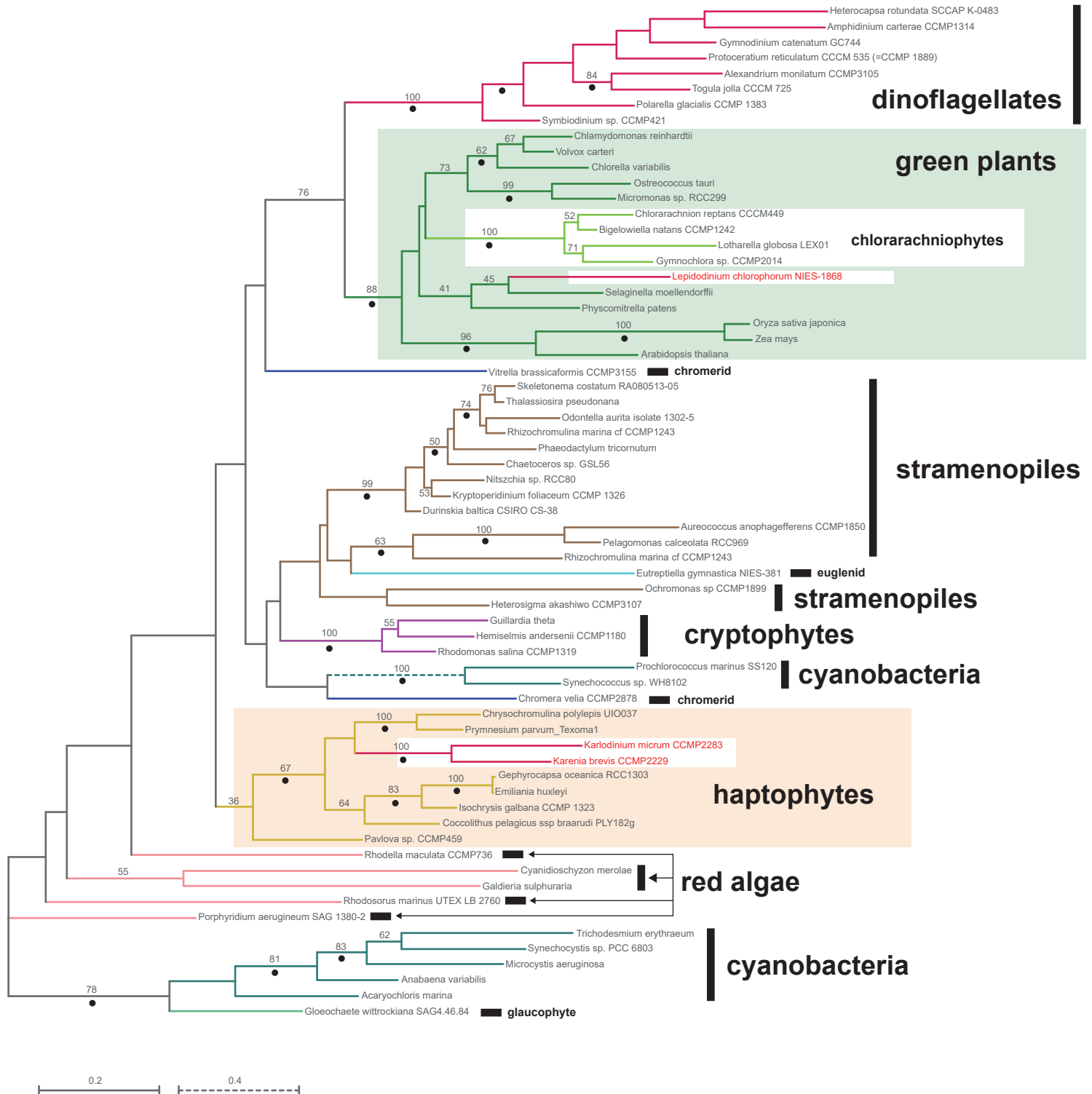

F-DVR

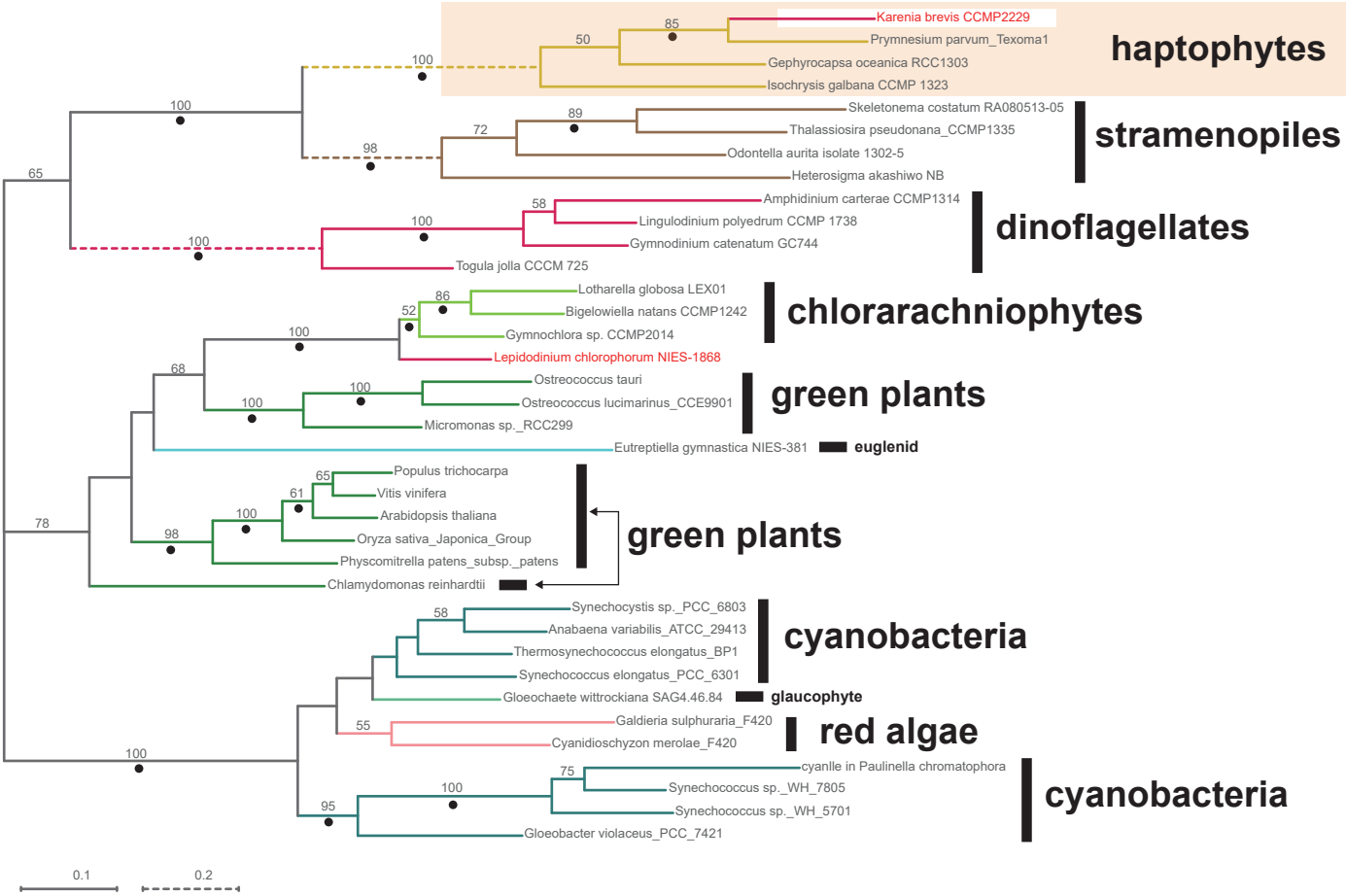

N-DVR

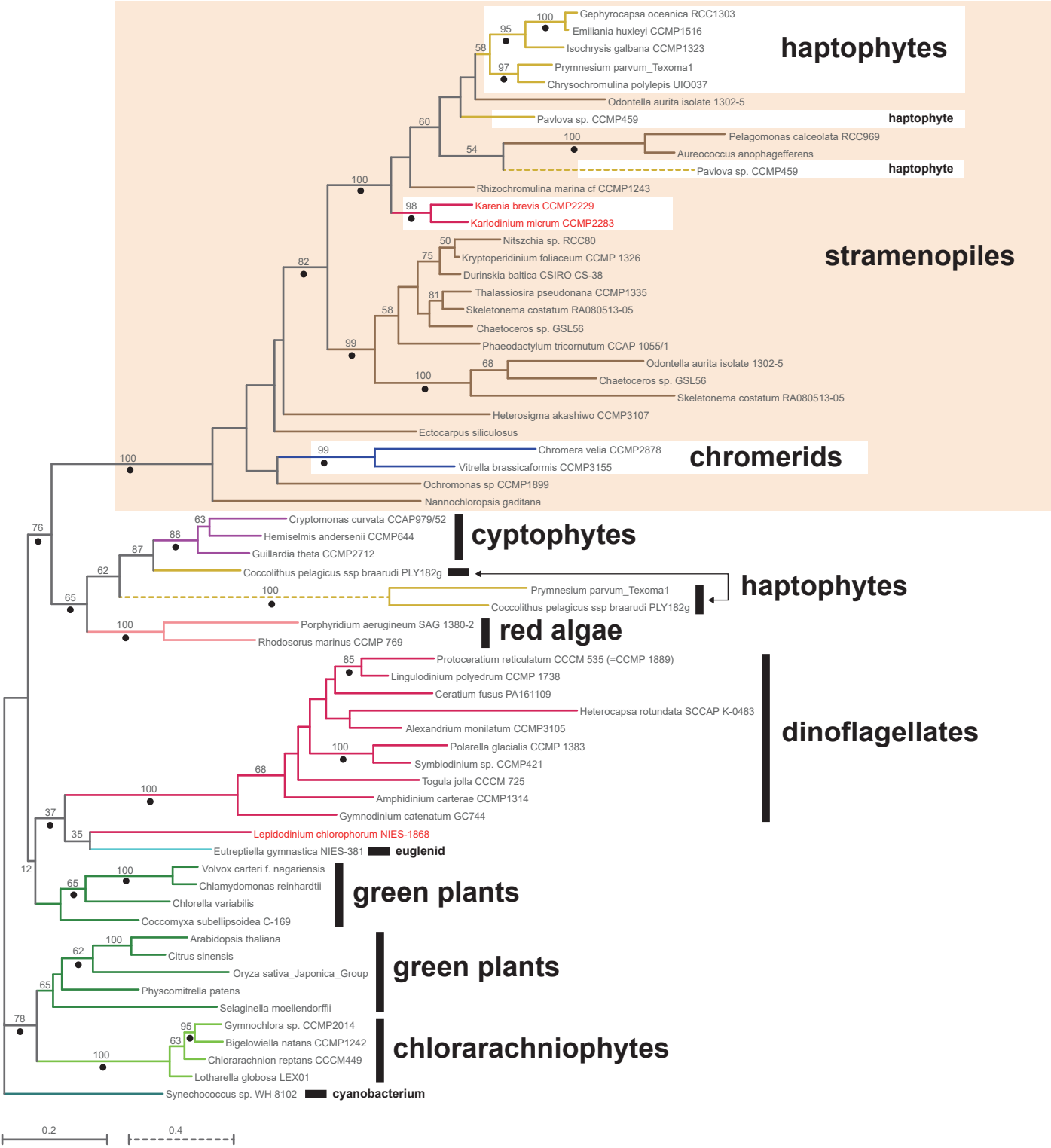

## POR (light-dependent)

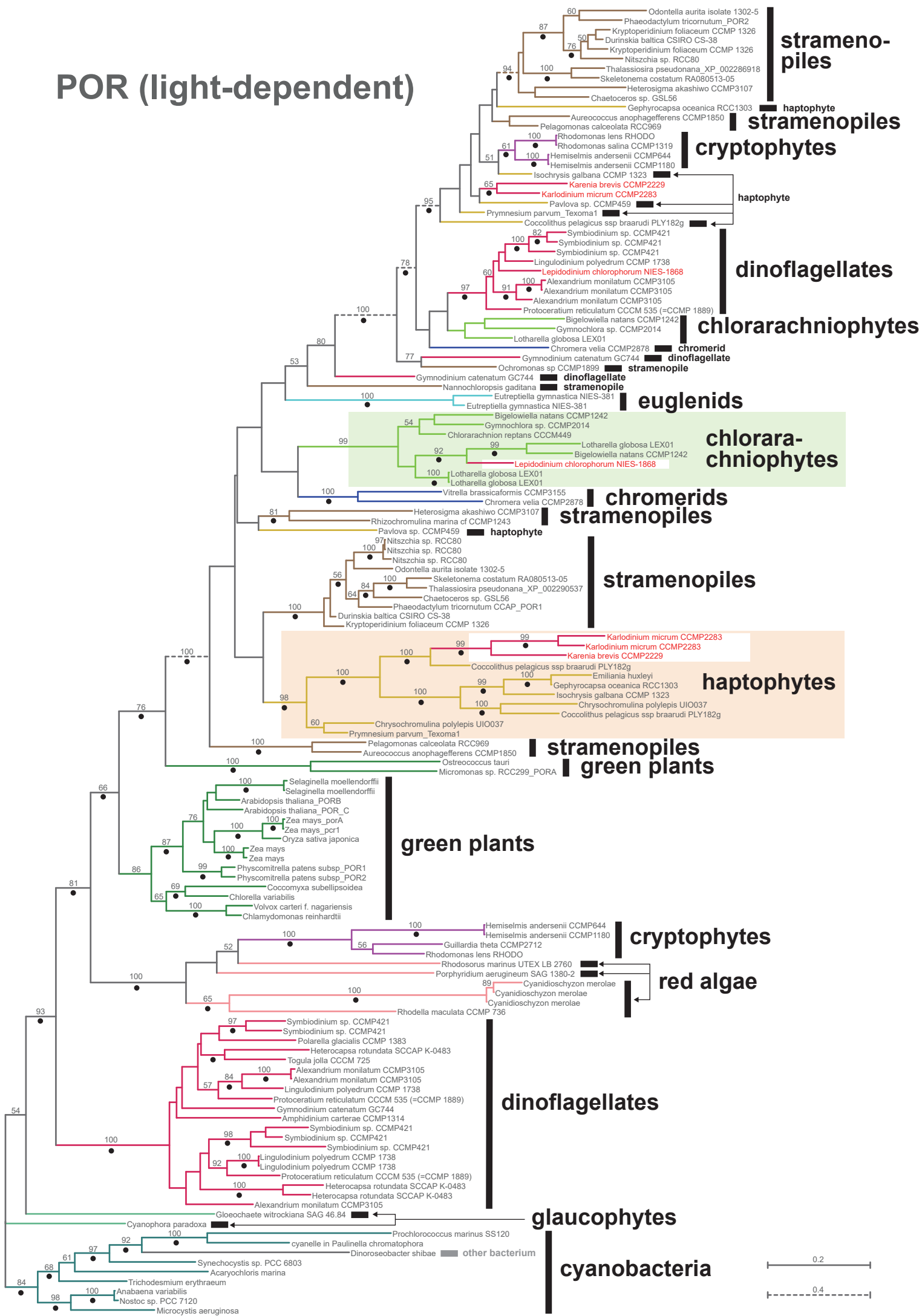

CS

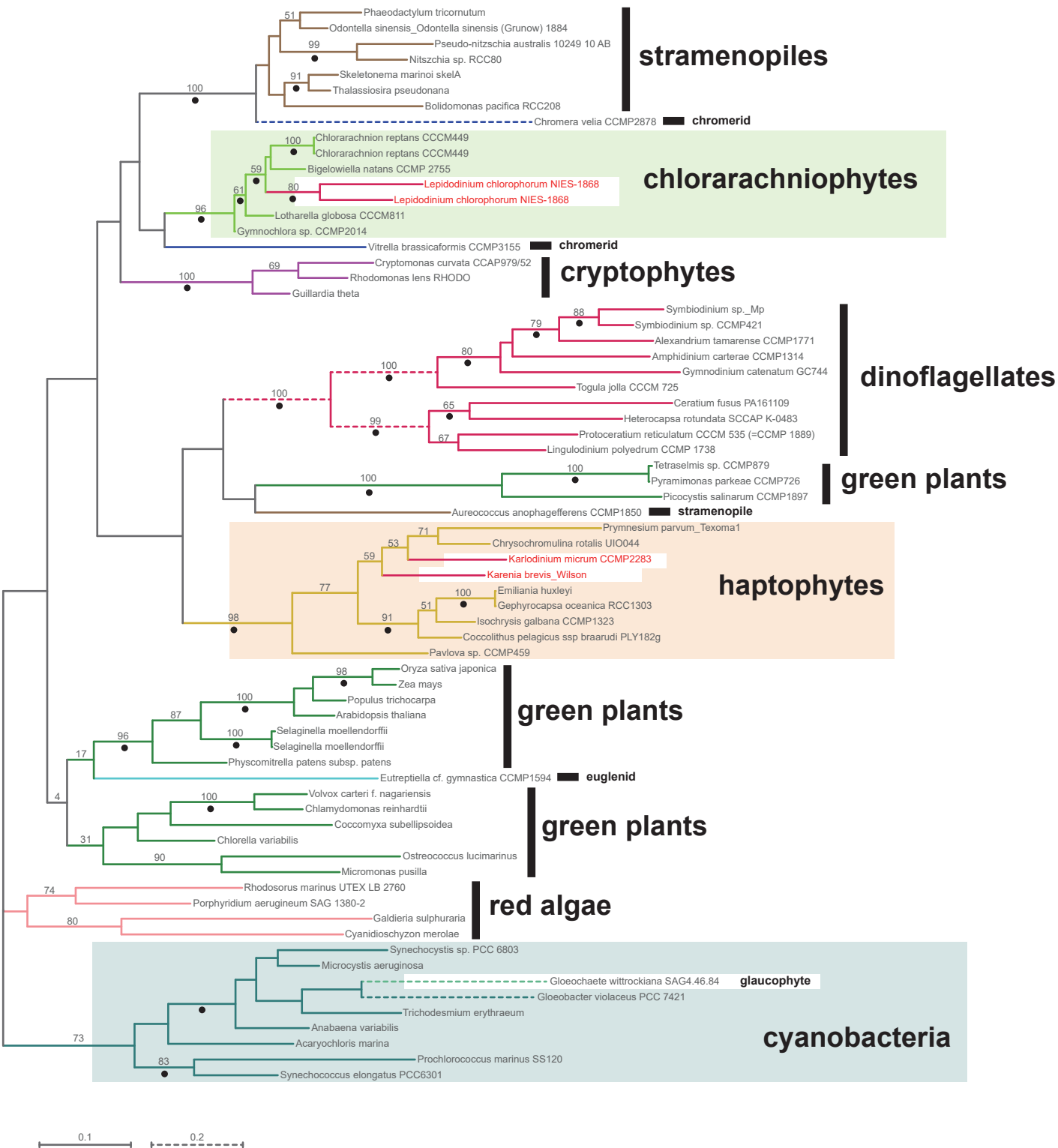

Supplement: Supplemental Information 2 — The details of the figures are same as those of Fig. 3, but full sequence names and their taxonomic classifications are indicated. [file peerj-06-5345-s002.pdf]
