# Supplementary material for: Patterns in evolutionary origins of heme, chlorophyll a and isopentenyl diphosphate biosynthetic pathways suggest non-photosynthetic periods prior to plastid replacements in dinoflagellates
Source: PeerJ. 2018 Aug 3;6:e5345. doi: 10.7717/peerj.5345 (PMC6078071; doi:10.7717/peerj.5345)
Supplement: Supplemental Information 3 — The details of the figures are same as those of Fig. 4, but full sequence names and their taxonomic classifications are indicated. [file peerj-06-5345-s003.pdf]

DXS

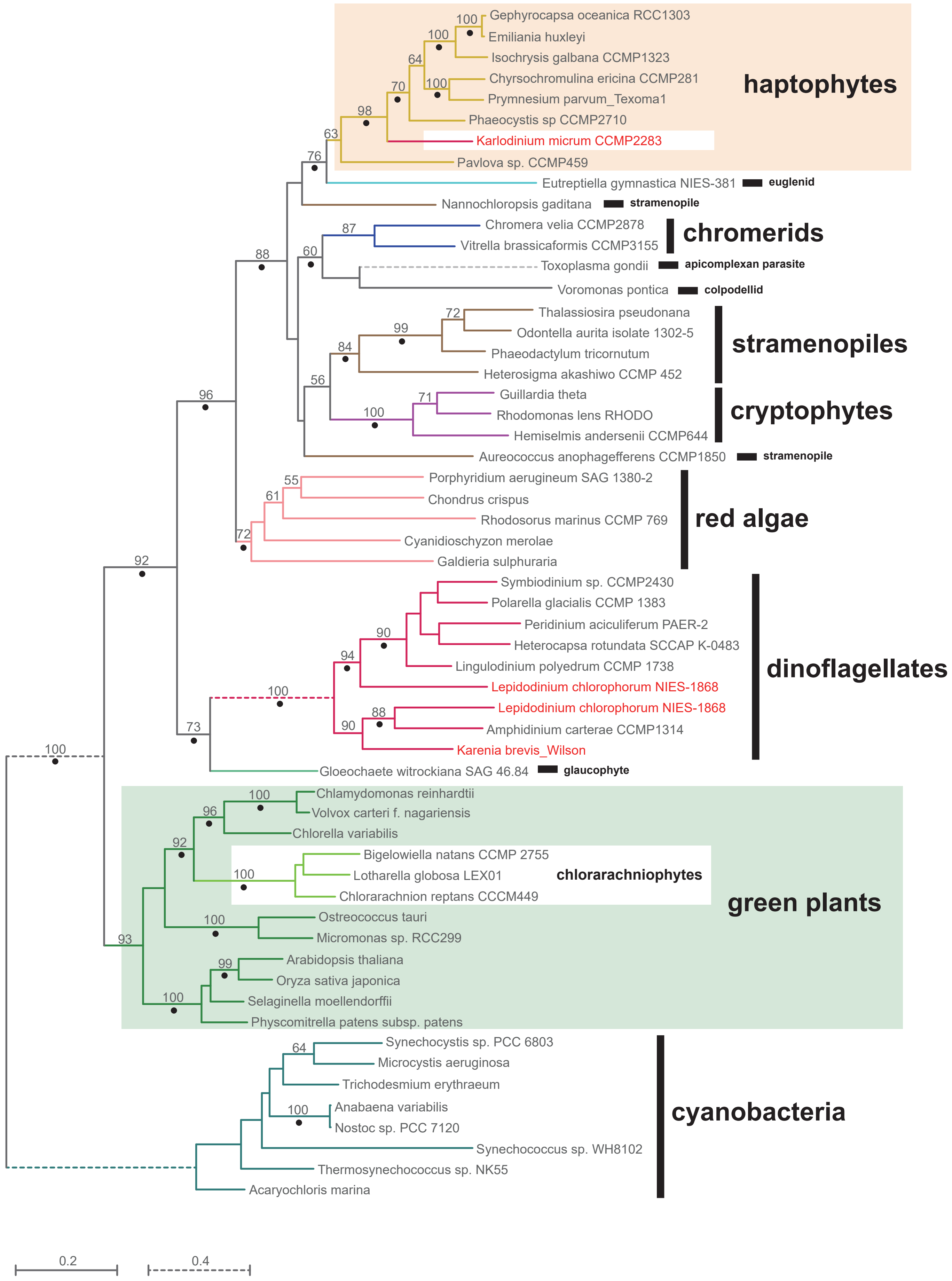

Gephyrocapsa oceanica RCC1303
Emiliania huxleyi
Isochrysis galbana CCMP1323
Chyrsochromulina ericina CCMP281
Prymnesium parvum_Texoma1
Phaeocystis sp CCMP2710
Karlodinium micrum CCMP2283
Pavlova sp. CCMP459
haptophytes
Eutreptiella gymnastica NIES-381
euglenid
Nannochloropsis gaditana
stramenopile
Chromera velia CCMP2878
Vitrella brassicaformis CCMP3155
chromerids
Toxoplasma gondii
apicomplexan parasite
Voromonas pontica
colpodellid
Thalassiosira pseudonana
Odontella aurita isolate 1302-5
Phaeodactylum tricornutum
Heterosigma akashiwo CCMP 452
stramenopiles
Guillardia theta
Rhodomonas lens RHODO
Hemiselmis andersenii CCMP644
cryptophytes
Aureococcus anophagefferens CCMP1850
stramenopile
Porphyridium aerugineum SAG 1380-2
Chondrus crispus
Rhodosorus marinus CCMP 769
Cyanidioschyzon merolae
Galdieria sulphuraria
red algae
Symbiodinium sp. CCMP2430
Polarella glacialis CCMP 1383
Peridinium aciculiferum PAER-2
Heterocapsa rotundata SCCAP K-0483
Lingulodinium polyedrum CCMP 1738
Lepidodinium chlorophorum NIES-1868
Lepidodinium chlorophorum NIES-1868
Amphidinium carterae CCMP1314
Karenia brevis_Wilson
dinoflagellates
Gloeochaete witrockiana SAG 46.84
glaucophyte
Chlamydomonas reinhardtii
Volvox carteri f. nagariensis
Chlorella variabilis
Bigelowiella natans CCMP 2755
Lotharella globosa LEX01
Chlorarachnion reptans CCCM449
chlorarachniophytes
Ostreococcus tauri
Micromonas sp. RCC299
Arabidopsis thaliana
Oryza sativa japonica
Selaginella moellendorffii
Physcomitrella patens subsp. patens
green plants
Synechocystis sp. PCC 6803
Microcystis aeruginosa
Trichodesmium erythraeum
Anabaena variabilis
Nostoc sp. PCC 7120
Synechococcus sp. WH8102
Thermosynechococcus sp. NK55
Acaryochloris marina
cyanobacteria
0.2
0.4

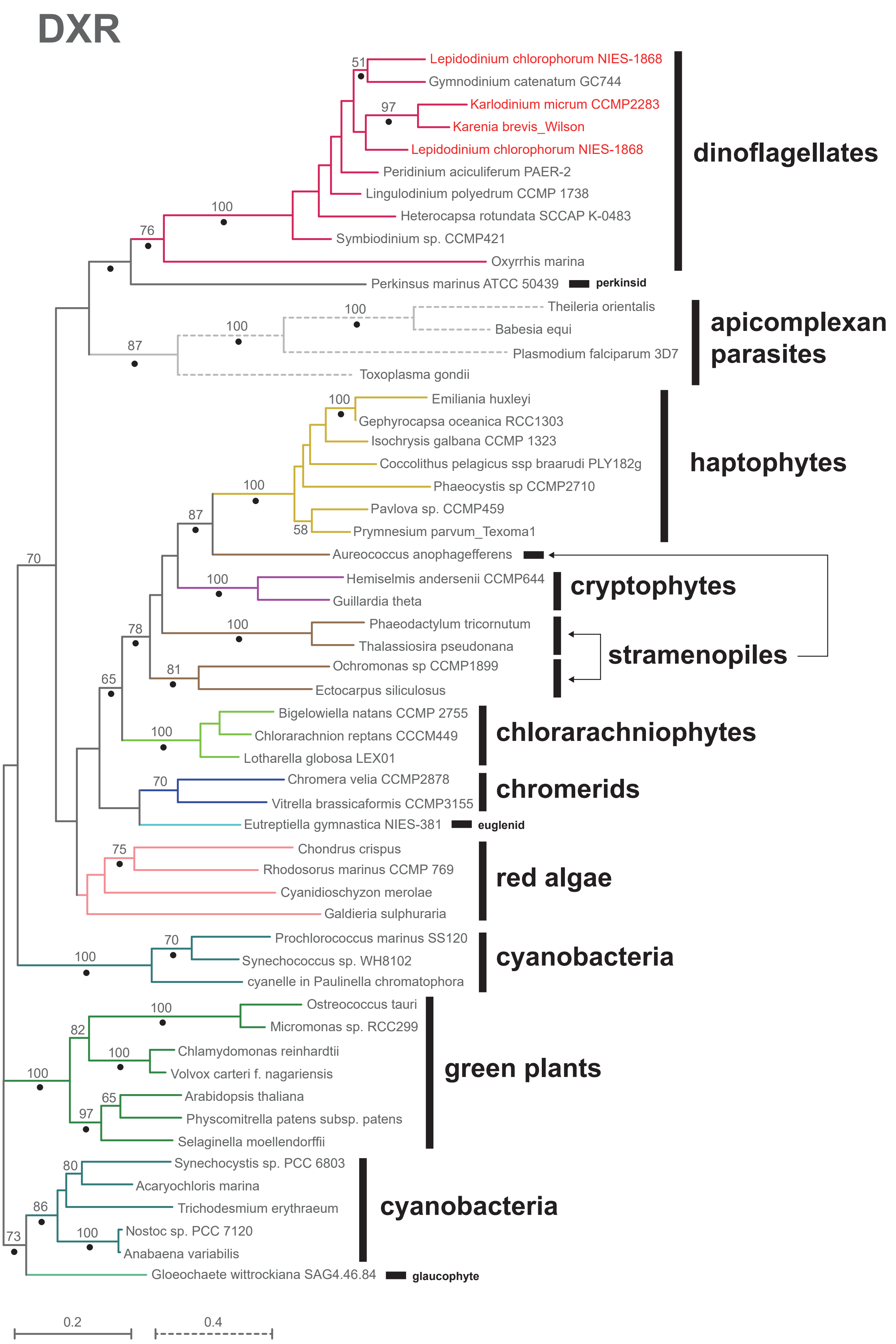

DXR
Lepidodinium chlorophorum NIES-1868
Gymnodinium catenatum GC744
Karlodinium micrum CCMP2283
Karenia brevis_Wilson
Lepidodinium chlorophorum NIES-1868
Peridinium aciculiferum PAER-2
Lingulodinium polyedrum CCMP 1738
Heterocapsa rotundata SCCAP K-0483
Symbiodinium sp. CCMP421
Oxyrrhis marina
Perkinsus marinus ATCC 50439
perkinsid
dinoflagellates
Theileria orientalis
Babesia equi
Plasmodium falciparum 3D7
Toxoplasma gondii
apicomplexan parasites
Emiliania huxleyi
Gephyrocapsa oceanica RCC1303
Isochrysis galbana CCMP 1323
Coccolithus pelagicus ssp braarudi PLY182g
Phaeocystis sp CCMP2710
Pavlova sp. CCMP459
Prymnesium parvum_Texoma1
haptophytes
Aureococcus anophagefferens
Hemiselmis andersenii CCMP644
Guillardia theta
cryptophytes
Phaeodactylum tricornutum
Thalassiosira pseudonana
Ochromonas sp CCMP1899
Ectocarpus siliculosus
stramenopiles
Bigelowiella natans CCMP 2755
Chlorarachnion reptans CCCM449
Lotharella globosa LEX01
chlorarachniophytes
Chromera velia CCMP2878
Vitrella brassicaformis CCMP3155
chromerids
Eutreptiella gymnastica NIES-381
euglenid
Chondrus crispus
Rhodosorus marinus CCMP 769
Cyanidioschyzon merolae
Galdieria sulphuraria
red algae
Prochlorococcus marinus SS120
Synechococcus sp. WH8102
cyanelle in Paulinella chromatophora
cyanobacteria
Ostreococcus tauri
Micromonas sp. RCC299
Chlamydomonas reinhardtii
Volvox carteri f. nagariensis
Arabidopsis thaliana
Physcomitrella patens subsp. patens
Selaginella moellendorffii
green plants
Synechocystis sp. PCC 6803
Acaryochloris marina
Trichodesmium erythraeum
Nostoc sp. PCC 7120
Anabaena variabilis
cyanobacteria
Gloeochaete wittrockiana SAG4.46.84
glaucophyte
0.2
0.4

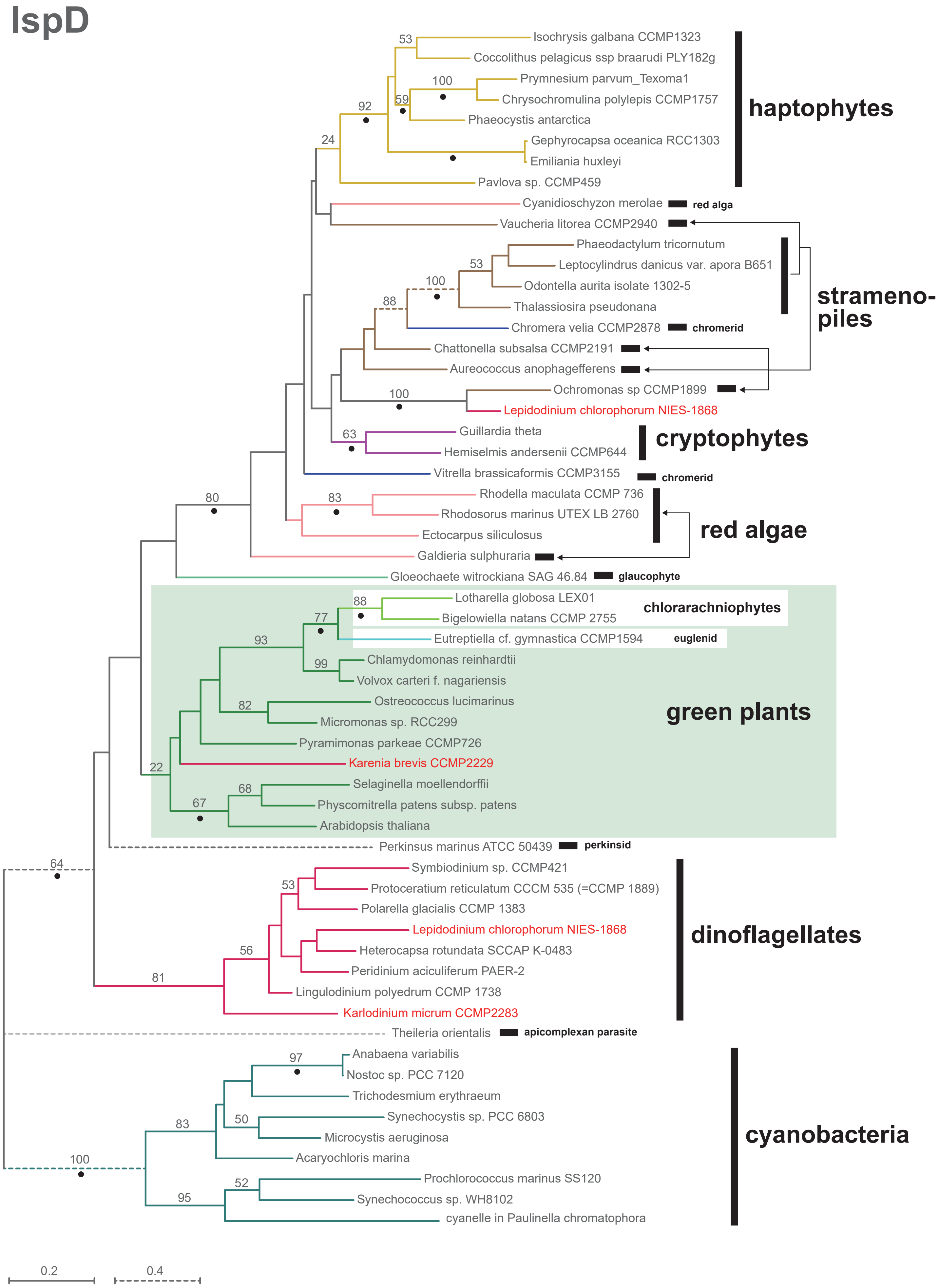
IspD
Isochrysis galbana CCMP1323
Coccolithus pelagicus ssp braarudi PLY182g
Prymnesium parvum_Texoma1
Chrysochromulina polylepis CCMP1757
Phaeocystis antarctica
Gephyrocapsa oceanica RCC1303
Emiliania huxleyi
Pavlova sp. CCMP459
haptophytes
Cyanidioschyzon merolae
red alga
Vaucheria litorea CCMP2940
Phaeodactylum tricornutum
Leptocylindrus danicus var. apora B651
Odontella aurita isolate 1302-5
Thalassiosira pseudonana
Chromera velia CCMP2878
chromerid
Chattonella subsalsa CCMP2191
Aureococcus anophagefferens
Ochromonas sp CCMP1899
Lepidodinium chlorophorum NIES-1868
strameno-piles
Guillardia theta
Hemiselmis andersenii CCMP644
cryptophytes
Vitrella brassicaformis CCMP3155
chromerid
Rhodella maculata CCMP 736
Rhodosorus marinus UTEX LB 2760
Ectocarpus siliculosus
Galdieria sulphuraria
red algae
Gloeochaete witrockiana SAG 46.84
glaucophyte
Lotharella globosa LEX01
Bigelowiella natans CCMP 2755
chlorarachniophytes
Eutreptiella cf. gymnastica CCMP1594
euglenid
Chlamydomonas reinhardtii
Volvox carteri f. nagariensis
Ostreococcus lucimarinus
Micromonas sp. RCC299
Pyramimonas parkeae CCMP726
Karenia brevis CCMP2229
Selaginella moellendorffii
Physcomitrella patens subsp. patens
Arabidopsis thaliana
green plants
Perkinsus marinus ATCC 50439
perkinsid
Symbiodinium sp. CCMP421
Protoceratium reticulatum CCCM 535 (=CCMP 1889)
Polarella glacialis CCMP 1383
Lepidodinium chlorophorum NIES-1868
Heterocapsa rotundata SCCAP K-0483
Peridinium aciculiferum PAER-2
Lingulodinium polyedrum CCMP 1738
Karlodinium micrum CCMP2283
dinoflagellates
Theileria orientalis
apicomplexan parasite
Anabaena variabilis
Nostoc sp. PCC 7120
Trichodesmium erythraeum
Synechocystis sp. PCC 6803
Microcystis aeruginosa
Acaryochloris marina
Prochlorococcus marinus SS120
Synechococcus sp. WH8102
cyanelle in Paulinella chromatophora
cyanobacteria
53
100
59
92
24
53
100
88
100
63
80
83
88
77
93
99
82
22
68
67
64
53
56
81
97
50
83
100
52
95
0.2
0.4

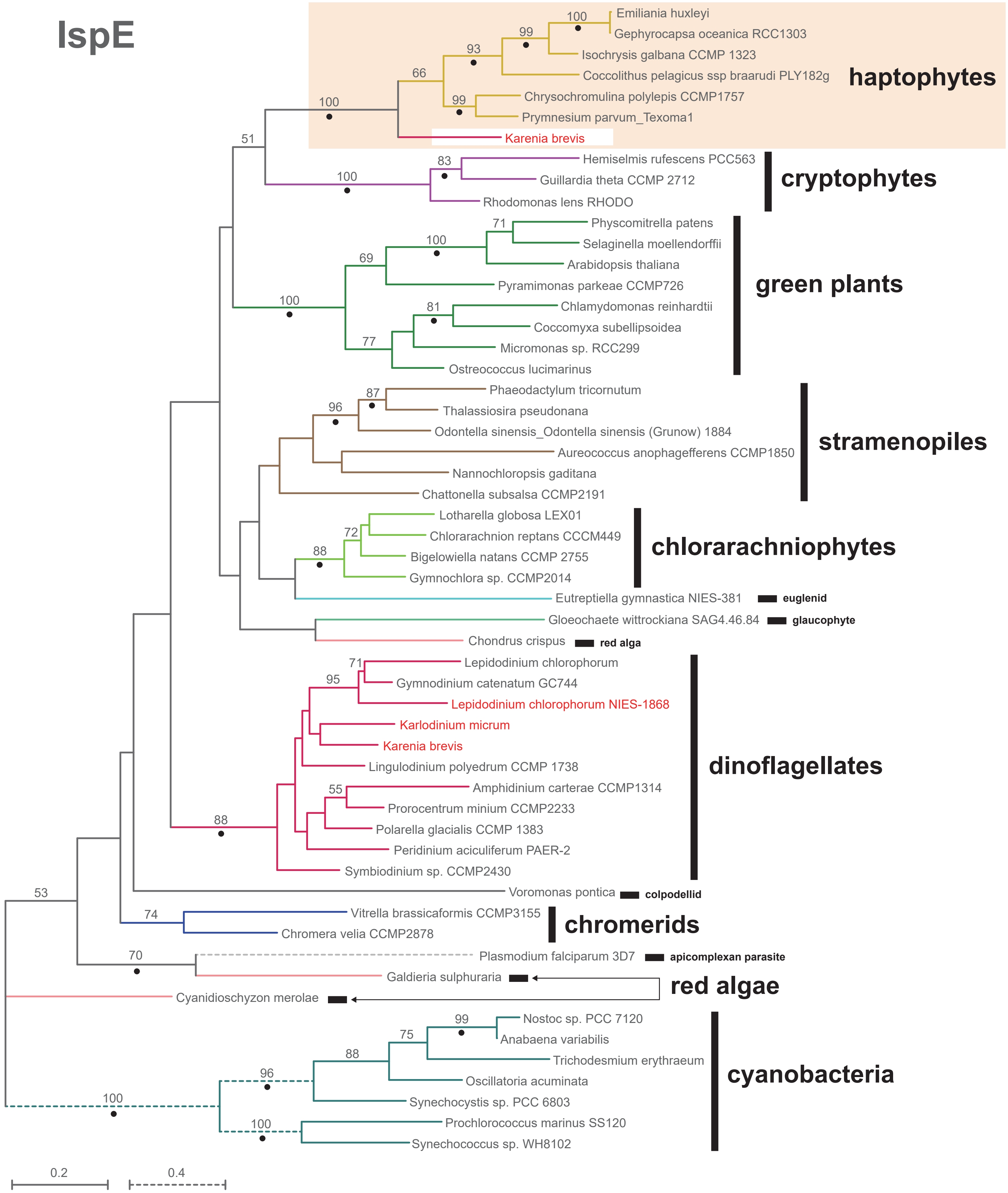

IspE
haptophytes
Emiliania huxleyi
Gephyrocapsa oceanica RCC1303
Isochrysis galbana CCMP 1323
Coccolithus pelagicus ssp braarudi PLY182g
Chrysochromulina polylepis CCMP1757
Prymnesium parvum_Texoma1
Karenia brevis
cryptophytes
Hemiselmis rufescens PCC563
Guillardia theta CCMP 2712
Rhodomonas lens RHODO
green plants
Physcomitrella patens
Selaginella moellendorffii
Arabidopsis thaliana
Pyramimonas parkeae CCMP726
Chlamydomonas reinhardtii
Coccomyxa subellipsoidea
Micromonas sp. RCC299
Ostreococcus lucimarinus
stramenopiles
Phaeodactylum tricornutum
Thalassiosira pseudonana
Odontella sinensis_Odontella sinensis (Grunow) 1884
Aureococcus anophagefferens CCMP1850
Nannochloropsis gaditana
Chattonella subsalsa CCMP2191
chlorarachniophytes
Lotharella globosa LEX01
Chlorarachnion reptans CCCM449
Bigelowiella natans CCMP 2755
Gymnochlora sp. CCMP2014
Eutreptiella gymnastica NIES-381
euglenid
Gloeochaete wittrockiana SAG4.46.84
glaucophyte
Chondrus crispus
red alga
dinoflagellates
Lepidodinium chlorophorum
Gymnodinium catenatum GC744
Lepidodinium chlorophorum NIES-1868
Karlodinium micrum
Karenia brevis
Lingulodinium polyedrum CCMP 1738
Amphidinium carterae CCMP1314
Prorocentrum minium CCMP2233
Polarella glacialis CCMP 1383
Peridinium aciculiferum PAER-2
Symbiodinium sp. CCMP2430
Voromonas pontica
colpodellid
chromerids
Vitrella brassicaformis CCMP3155
Chromera velia CCMP2878
Plasmodium falciparum 3D7
apicomplexan parasite
Galdieria sulphuraria
Cyanidioschyzon merolae
red algae
cyanobacteria
Nostoc sp. PCC 7120
Anabaena variabilis
Trichodesmium erythraeum
Oscillatoria acuminata
Synechocystis sp. PCC 6803
Prochlorococcus marinus SS120
Synechococcus sp. WH8102
0.2
0.4

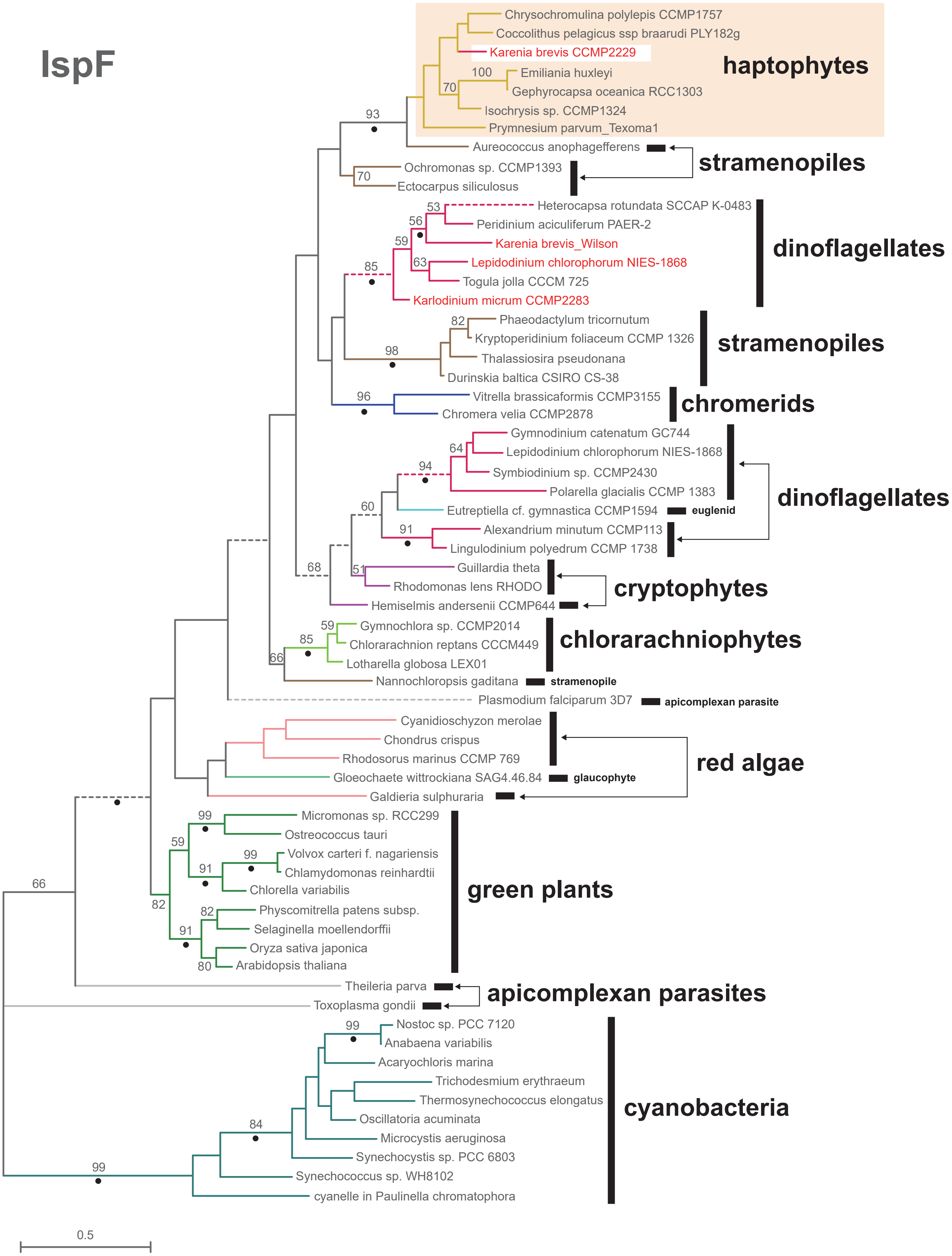
IspF
haptophytes
Chrysochromulina polylepis CCMP1757
Coccolithus pelagicus ssp braarudi PLY182g
Karenia brevis CCMP2229
Emiliania huxleyi
Gephyrocapsa oceanica RCC1303
Isochrysis sp. CCMP1324
Prymnesium parvum_Texoma1
Aureococcus anophagefferens
stramenopiles
Ochromonas sp. CCMP1393
Ectocarpus siliculosus
Heterocapsa rotundata SCCAP K-0483
Peridinium aciculiferum PAER-2
Karenia brevis_Wilson
Lepidodinium chlorophorum NIES-1868
Togula jolla CCCM 725
Karlodinium micrum CCMP2283
dinoflagellates
Phaeodactylum tricornutum
Kryptoperidinium foliaceum CCMP 1326
Thalassiosira pseudonana
Durinskia baltica CSIRO CS-38
stramenopiles
Vitrella brassicaformis CCMP3155
Chromera velia CCMP2878
chromerids
Gymnodinium catenatum GC744
Lepidodinium chlorophorum NIES-1868
Symbiodinium sp. CCMP2430
Polarella glacialis CCMP 1383
Eutreptiella cf. gymnastica CCMP1594
euglenid
Alexandrium minutum CCMP113
Lingulodinium polyedrum CCMP 1738
dinoflagellates
Guillardia theta
Rhodomonas lens RHODO
Hemiselmis andersenii CCMP644
cryptophytes
Gymnochlora sp. CCMP2014
Chlorarachnion reptans CCCM449
Lotharella globosa LEX01
chlorarachniophytes
Nannochloropsis gaditana
stramenopile
Plasmodium falciparum 3D7
apicomplexan parasite
Cyanidioschyzon merolae
Chondrus crispus
Rhodosorus marinus CCMP 769
Gloeochaete wittrockiana SAG4.46.84
glaucophyte
Galdieria sulphuraria
red algae
Micromonas sp. RCC299
Ostreococcus tauri
Volvox carteri f. nagariensis
Chlamydomonas reinhardtii
Chlorella variabilis
Physcomitrella patens subsp.
Selaginella moellendorffii
Oryza sativa japonica
Arabidopsis thaliana
green plants
Theileria parva
Toxoplasma gondii
apicomplexan parasites
Nostoc sp. PCC 7120
Anabaena variabilis
Acaryochloris marina
Trichodesmium erythraeum
Thermosynechococcus elongatus
Oscillatoria acuminata
Microcystis aeruginosa
Synechocystis sp. PCC 6803
Synechococcus sp. WH8102
cyanelle in Paulinella chromatophora
cyanobacteria
0.5

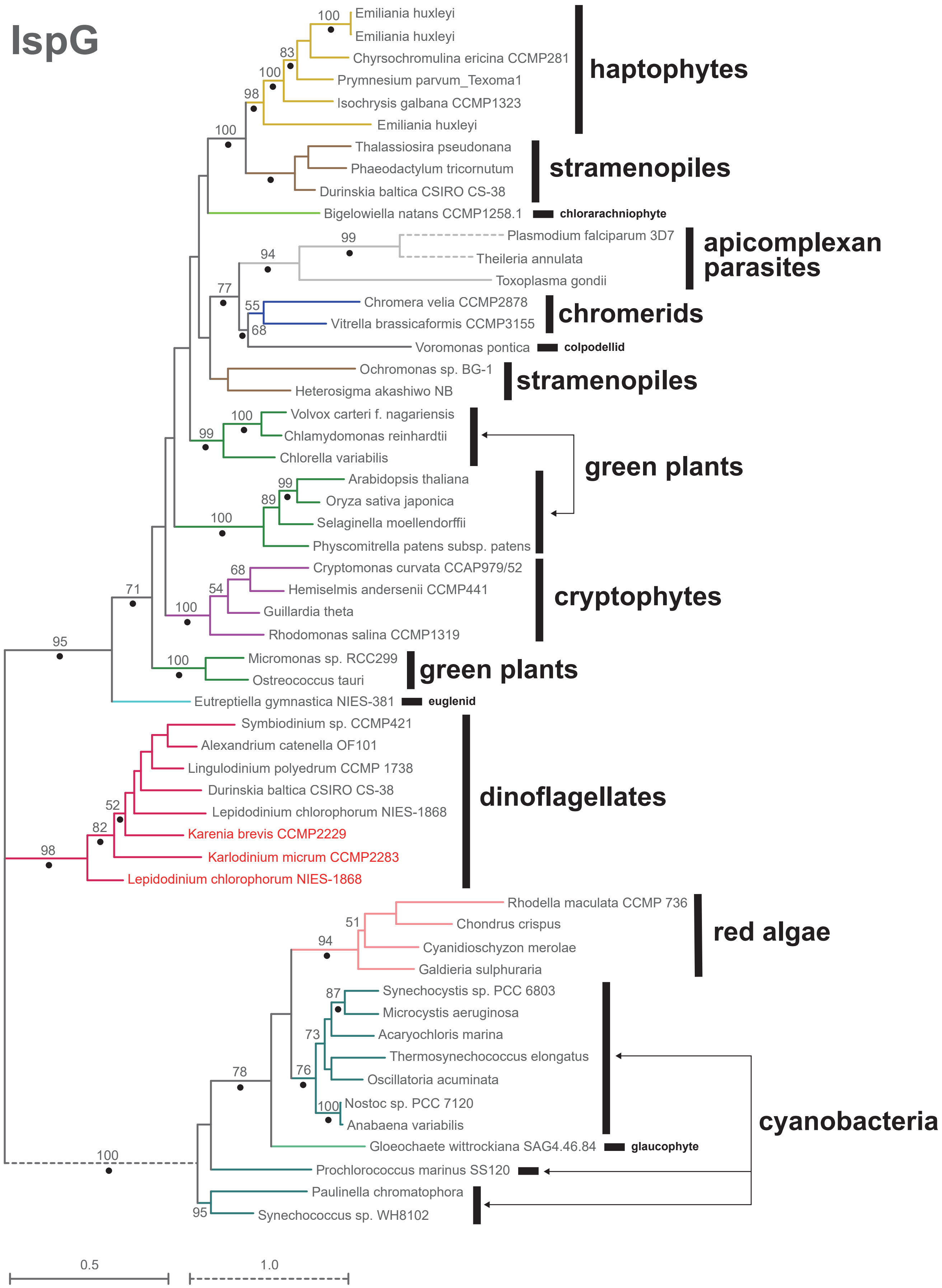
IspG
Emiliania huxleyi
Emiliania huxleyi
Chyrsochromulina ericina CCMP281
Prymnesium parvum_Texoma1
Isochrysis galbana CCMP1323
Emiliania huxleyi
haptophytes
Thalassiosira pseudonana
Phaeodactylum tricornutum
Durinskia baltica CSIRO CS-38
stramenopiles
Bigelowiella natans CCMP1258.1
chlorarachniophyte
Plasmodium falciparum 3D7
Theileria annulata
Toxoplasma gondii
apicomplexan parasites
Chromera velia CCMP2878
Vitrella brassicaformis CCMP3155
chromerids
Voromonas pontica
colpodellid
Ochromonas sp. BG-1
Heterosigma akashiwo NB
stramenopiles
Volvox carteri f. nagariensis
Chlamydomonas reinhardtii
Chlorella variabilis
green plants
Arabidopsis thaliana
Oryza sativa japonica
Selaginella moellendorffii
Physcomitrella patens subsp. patens
Cryptomonas curvata CCAP979/52
Hemiselmis andersenii CCMP441
Guillardia theta
Rhodomonas salina CCMP1319
cryptophytes
Micromonas sp. RCC299
Ostreococcus tauri
green plants
Eutreptiella gymnastica NIES-381
euglenid
Symbiodinium sp. CCMP421
Alexandrium catenella OF101
Lingulodinium polyedrum CCMP 1738
Durinskia baltica CSIRO CS-38
Lepidodinium chlorophorum NIES-1868
Karenia brevis CCMP2229
Karlodinium micrum CCMP2283
Lepidodinium chlorophorum NIES-1868
dinoflagellates
Rhodella maculata CCMP 736
Chondrus crispus
Cyanidioschyzon merolae
Galdieria sulphuraria
red algae
Synechocystis sp. PCC 6803
Microcystis aeruginosa
Acaryochloris marina
Thermosynechococcus elongatus
Oscillatoria acuminata
Nostoc sp. PCC 7120
Anabaena variabilis
Gloeochaete wittrockiana SAG4.46.84
glaucophyte
Prochlorococcus marinus SS120
Paulinella chromatophora
Synechococcus sp. WH8102
cyanobacteria
100
83
100
98
100
99
94
77
55
68
100
99
99
89
100
68
54
100
71
95
100
52
82
98
51
94
87
73
76
100
78
100
95
0.5
1.0

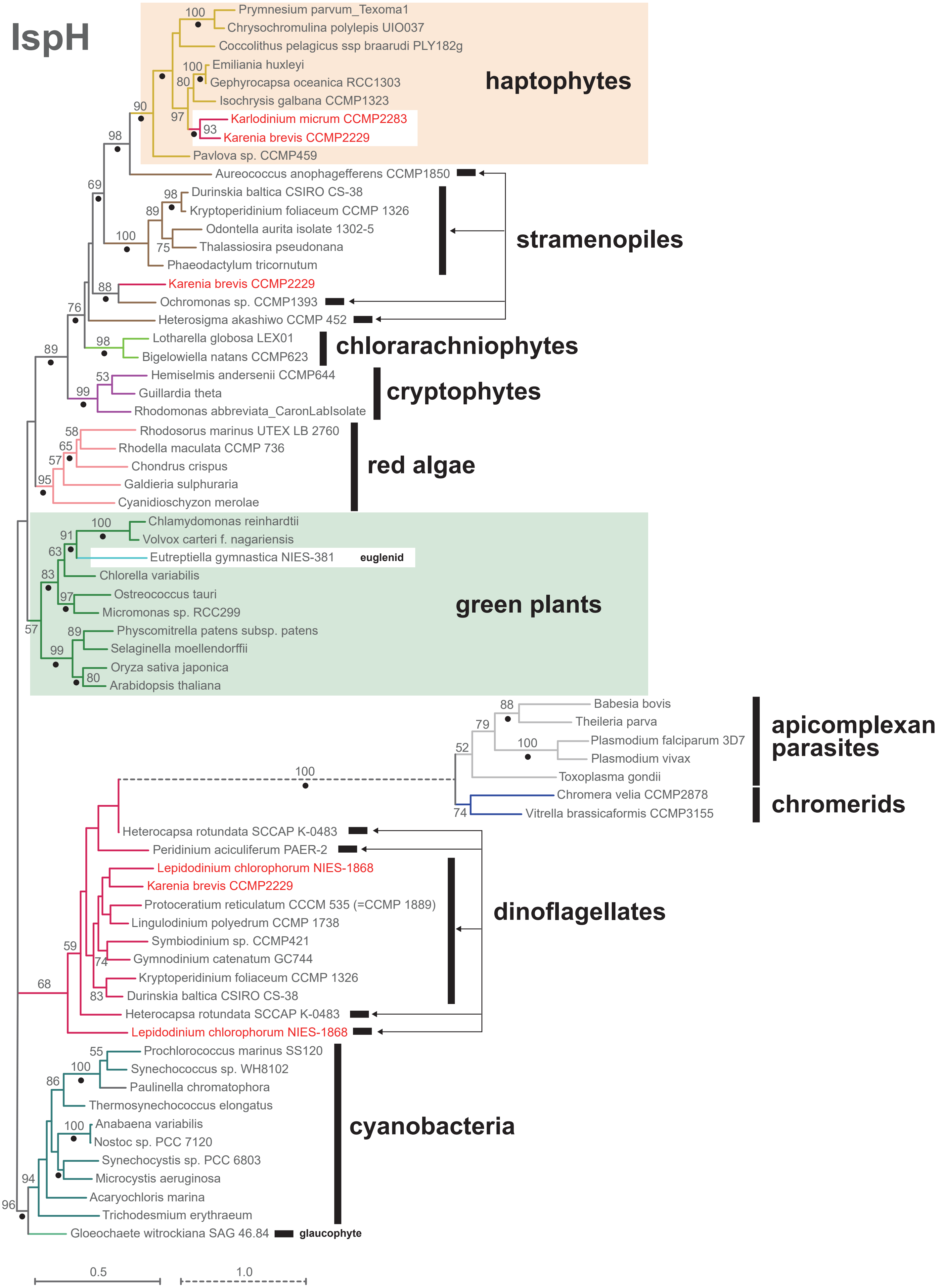

IspH
haptophytes
Prymnesium parvum_Texoma1
Chrysochromulina polylepis UIO037
Coccolithus pelagicus ssp braarudi PLY182g
Emiliania huxleyi
Gephyrocapsa oceanica RCC1303
Isochrysis galbana CCMP1323
Karlodinium micrum CCMP2283
Karenia brevis CCMP2229
Pavlova sp. CCMP459
Aureococcus anophagefferens CCMP1850
stramenopiles
Durinskia baltica CSIRO CS-38
Kryptoperidinium foliaceum CCMP 1326
Odontella aurita isolate 1302-5
Thalassiosira pseudonana
Phaeodactylum tricornutum
Karenia brevis CCMP2229
Ochromonas sp. CCMP1393
Heterosigma akashiwo CCMP 452
chlorarachniophytes
Lotharella globosa LEX01
Bigelowiella natans CCMP623
cryptophytes
Hemiselmis andersenii CCMP644
Guillardia theta
Rhodomonas abbreviata_CaronLabIsolate
red algae
Rhodosorus marinus UTEX LB 2760
Rhodella maculata CCMP 736
Chondrus crispus
Galdieria sulphuraria
Cyanidioschyzon merolae
green plants
Chlamydomonas reinhardtii
Volvox carteri f. nagariensis
Eutreptiella gymnastica NIES-381
euglenid
Chlorella variabilis
Ostreococcus tauri
Micromonas sp. RCC299
Physcomitrella patens subsp. patens
Selaginella moellendorffii
Oryza sativa japonica
Arabidopsis thaliana
apicomplexan parasites
Babesia bovis
Theileria parva
Plasmodium falciparum 3D7
Plasmodium vivax
Toxoplasma gondii
chromerids
Chromera velia CCMP2878
Vitrella brassicaformis CCMP3155
dinoflagellates
Heterocapsa rotundata SCCAP K-0483
Peridinium aciculiferum PAER-2
Lepidodinium chlorophorum NIES-1868
Karenia brevis CCMP2229
Protoceratium reticulatum CCCM 535 (=CCMP 1889)
Lingulodinium polyedrum CCMP 1738
Symbiodinium sp. CCMP421
Gymnodinium catenatum GC744
Kryptoperidinium foliaceum CCMP 1326
Durinskia baltica CSIRO CS-38
Heterocapsa rotundata SCCAP K-0483
Lepidodinium chlorophorum NIES-1868
cyanobacteria
Prochlorococcus marinus SS120
Synechococcus sp. WH8102
Paulinella chromatophora
Thermosynechococcus elongatus
Anabaena variabilis
Nostoc sp. PCC 7120
Synechocystis sp. PCC 6803
Microcystis aeruginosa
Acaryochloris marina
Trichodesmium erythraeum
Gloeochaete witrockiana SAG 46.84
glaucophyte
0.5
1.0
